# Characterization and heterologous expression of the neoabyssomicin/abyssomicin biosynthetic gene cluster from *Streptomyces koyangensis* SCSIO 5802

**DOI:** 10.1186/s12934-018-0875-1

**Published:** 2018-02-20

**Authors:** Jiajia Tu, Siting Li, Jiang Chen, Yongxiang Song, Shaobin Fu, Jianhua Ju, Qinglian Li

**Affiliations:** 1 CAS Key Laboratory of Tropical Marine Bio-resources and Ecology, Guangdong Key Laboratory of Marine Materia Medica, RNAM Center for Marine Microbiology, South China Sea Institute of Oceanology, Chinese Academy of Sciences, 164 West Xingang Road, Guangzhou, 510301 China; 2 School of Pharmacy, Zunyi Medical University, 201 Dalian Road, Zunyi, 563000 China; 3 College of Bio and Marine Sciences, Shenzhen University, 3688 Nanhai Ave, Shenzhen, 518060 China; 4 University of Chinese Academy of Sciences, 19 Yuquan Road, Beijing, 110039 China

**Keywords:** Abyssomicin, Tetronate, Biosynthesis, Transporter, Pathway-specific regulator

## Abstract

**Background:**

The deep-sea-derived microbe *Streptomyces koyangensis* SCSIO 5802 produces neoabyssomicins A–B (**1**–**2**) and abyssomicins 2 (**3**) and 4 (**4**). Neoabyssomicin A (**1**) augments human immunodeficiency virus-1 (HIV-1) replication whereas abyssomicin 2 (**3**) selectively reactivates latent HIV and is also active against Gram-positive pathogens including methicillin-resistant *Staphylococcus aureus* (MRSA). Structurally, neoabyssomicins A–B constitute a new subtype within the abyssomicin family and feature unique structural traits characteristic of extremely interesting biosynthetic transformations.

**Results:**

In this work, the biosynthetic gene cluster (BGC) for the neoabyssomicins and abyssomicins, composed of 28 opening reading frames, was identified in *S. koyangensis* SCSIO 5802, and its role in neoabyssomicin/abyssomicin biosynthesis was confirmed via gene inactivation and heterologous expression experiments. Bioinformatics and genomics analyses enabled us to propose a biosynthetic pathway for neoabyssomicin/abyssomicin biosynthesis. Similarly, a protective export system by which both types of compounds are secreted from the *S. koyangensis* producer was identified, as was a four-component ABC transporter-based import system central to neoabyssomicin/abyssomicin biosynthesis. Furthermore, two regulatory genes, *abmI* and *abmH*, were unambiguously shown to be positive regulators of neoabyssomicin/abyssomicin biosynthesis. Consistent with their roles as positive regulatory genes, the overexpression of *abmI* and *abmH* (independent of each other) was shown to improve neoabyssomicin/abyssomicin titers.

**Conclusions:**

These studies provide new insight into the biosynthesis of the abyssomicin class of natural products, and highlight important exploitable features of its BGC for future efforts. Elucidation of the neoabyssomicin/abyssomicin BGC now enables combinatorial biosynthetic initiatives aimed at improving both the titers and pharmaceutical properties of these important natural products-based drug leads.

**Electronic supplementary material:**

The online version of this article (10.1186/s12934-018-0875-1) contains supplementary material, which is available to authorized users.

## Background

**Fig. 1:**
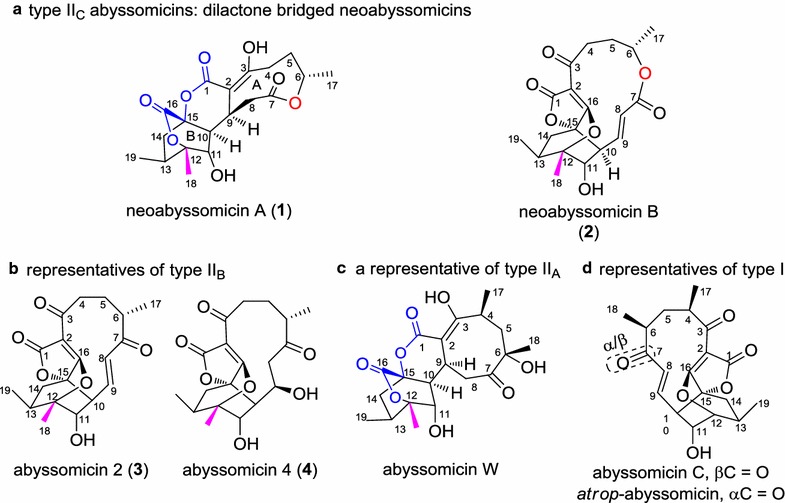
Chemical structures of representatives for each type/subtype of abyssomicin class of natural products. **a** Representatives for type II_C_ abyssomicins: neoabyssomicins A–B (**1**–**2**). **b** Representatives for type II_B_ abyssomicins: abyssomicin 2 (**3**) and 4 (**4**). **c** A representative for type II_A_ abyssomicins: abyssomicin W. **d** Representatives for type I abyssomicins: abyssomicin C and *atrop*-abyssomicin C

Infectious diseases constitute a leading cause of death worldwide and continue to advance in their lethality as drug resistance becomes more widespread. This concern, coupled with revelations about as yet, untapped sources of molecular diversity, has spurred intense efforts to discover new drug candidates with novel structures/modes of action able to circumvent or ablate bacterial mechanisms of drug resistance. Within this context, three novel natural products, abyssomicin C (Fig. 1) and its analogues abyssomicins B and D, were discovered from the marine actinomycete strain *Verrucosispora* sp. AB-18032 in 2004 [1]. Abyssomicin C exhibits promising antibacterial activities against a number of Gram-positive bacteria, including methicillin-resistant *Staphylococcus aureus* (MRSA) [2] and *Mycobacterium tuberculosis* [3]. Motivated in part by these important revelations, biosynthetic studies and bacterial activity screening programs have recently unveiled several new members of the abyssomicin family [4–10]. The abyssomicin class of natural products contains two related subfamilies (type I and type II) [10]. Structurally, type II family members are “enantiomeric” counterparts of the type I family compounds and are further grouped into two subtypes (type II_A_ and type II_B_) based on difference in C-4 methyl substitution (see Fig. 1 for the chemical structures of the representative for each type/subtype).

Recently, as part of our efforts to discover novel secondary metabolites with antibacterial activities from under-explored marine actinomycetes, two new analogues of the abyssomicin class, neoabyssomicins A–B (**1**–**2**) along with abyssomicins 2 (**3**) and 4 (**4**) (Fig. 1), were discovered from the deep-sea bacterium *Streptomyces koyangensis* SCSIO 5802 [10]. Neoabyssomicin A (**1**) augments HIV-1 replication in a human lymphocyte model. Notably, abyssomicin 2 (**3**) not only selectively reactivates latent HIV [9] but is also active against a panel of Gram-positive pathogens, including clinical methicillin-resistant (MRSA) strains, with MICs of 3–15 μg/mL [10]. Structurally, neoabyssomicins A–B constitute a new subtype (type II_C_) of the abyssomicins, featuring an inserted oxygen atom within the polyketide chain. Neoabyssomicin A (**1**) possesses a novel skeleton featuring a rare caged 6/6/6 ring system fused with two additional 6/9 lactone rings (Fig. 1). Neoabyssomicin B (**2**) has a 12-membered lactone ring in place of the 11-membered polyketide ring (Fig. 1). Both the unique structures and biological activities of neoabyssomicins/abyssomicins provide clear inspiration for understanding the biosynthetic mechanisms leading to their production as well as possible application of combinatorial biosynthesis to enhance yields and create structural diversity within the class.

A model for the biosynthesis of *atrop*-abyssomicin C has been proposed based on a combination of feeding studies with ^13^C-labelled biosynthetic precursors and identification of its BGC from *Verrucosispora* sp. AB-18032 [11]. AbyU has been demonstrated to be a Diels–Alderase that catalyzes [4 + 2] cycloaddition to form a key cyclohexene in *atrop*-abyssomicin C [12]. Recently, a BGC, identified in *Streptomyces* sp. LC-6-2 by whole genome sequencing, was proposed to account for abyssomicin M–X biosynthesis [8]. Relative to *atrop*-abyssomicin C, neoabyssomicins A–B are characterized by a number of biosynthetically interesting structural features. Here, we report the application of genetics experiments and bioinformatics analyses to decipher the biosynthetic pathway leading to the neoabyssomicins/abyssomicins in *S. koyangensis* SCSIO 5802 as well as their transport systems. Furthermore, rationally designed enhancements to neoabyssomicin/abyssomicin production were realized via overexpression of two different pathway-specific positive regulators.

## Methods

### Bacterial strains, plasmids and culture conditions

The bacterial strains and plasmids are listed in Additional file 1: Tables S1 and S2, respectively. The culture conditions for *S. koyangensis* SCSIO 5802 has been previously described [10]. *Escherichia coli* including XL 1-blue MR, ET12567/pUZ8002, BW25113/pIJ790, as well as, culture conditions have also been previously described [13]. When necessary, antibiotics were supplemented at the following concentrations: apramycin (Apr) 50 μg/mL, chloramphenicol (Chl) 25 μg/mL, and kanamycin (Kan) 50 μg/mL.

### Complete genome sequencing and bioinformatics analysis

High-molecular-weight DNA of *S. koyangensis* SCSIO 5802 was isolated according to a slightly modified protocol [14]. Sequencing of the complete genome was accomplished using a combination of PacBio RSII sequencing (Pacific Biosciences) and Illumina Hiseq 2500 technologies at Biozeron Biotech Co., LTD (Shanghai, China). Genes involved in secondary metabolic pathways were predicted using online antiSMASH software (http://antismash.secondarymetabolites.org/). The deduced ORFs were analyzed using online FramePlot 4.0beta software (http://nocardia.nih.go.jp/fp4/) and their functional predictions were accomplished with an online BLAST program (http://blast.ncbi.nlm.nih.gov/). The PKS architectures were analyzed using an NRPS-PKS online website (http://nrps.igs.umaryland.edu/nrps/). The nucleotide sequence of *abm* BGC was deposited at GenBank under accession number MG243704.

### Construction of genomic cosmid library and inactivation of *S. koyangensis* SCSIO 5802 genes

The genomic cosmid library of *S. koyangensis* SCSIO 5802 was constructed using SuperCos1 according to the manufacturer’s protocol provided by the vector kit (Agilent). About 2000 clones were picked and placed into 96-well plates and stored at − 80 °C. We set out to probe the neoabyssomicin/abyssomicin BGC with three pairs of primers associated with *abmB1*, *orf(*−*2)* and *orf(*+*3)* (Additional file 1: Table S3) using PCR methods. These three designed primers were utilized to screen the picked 2000 clones, and 6 positive cosmids (10-8B, 9-7C, 7-6F, 21-9A, 6-7D and 29-8D) were obtained.

The λ-RED-mediated PCR-targeting mutagenesis method was then employed to inactivate targeted neoabyssomicin/abyssomicin biosynthetic genes [15]. Three cosmids 7-6F, 9-7C and 21-9A covering the whole neoabyssomicin/abyssomicin gene cluster were used to inactivate the genes in the neoabyssomicin/abyssomicin BGC. Primers designed for gene-specific inactivation are listed in Additional file 1: Table S3. An example for *abmB1* is detailed here. Cosmid 9-7C was introduced into *E. coli* BW25113/pIJ790 to inactivate *abmB1*. The *aac(3)IV*-*oriT* cassette was amplified by PCR from pIJ773 using primers abmB1-Del-F and abmB1-Del-R (Additional file 1: Table S3), and introduced into *E. coli* BW25113/pIJ790/cosmid 9-7C by electroporation to replace *abmB1* via λ-RED-mediated recombination. Correct recombination was established by PCR using primers abmB1-TF and abmB1-TR. The mutated cosmid 9-7C-ΔabmB1 was then introduced into *E. coli* ET12567/pUZ8002 for further conjugation with *S. koyangensis* SCSIO 5802. The double crossover mutant was obtained by antibiotic selection (Apr^R^Kan^S^) and confirmed by PCR using primers abmB1-TF and abmB1-TR.

### Construction of genomic PAC library and heterologous expression of the *abm* gene cluster

PAC library construction was performed using pESAC13-A by Bio S&T (Montreal, Canada). pESAC13-A was developed from pPAC-S1 [16] by Sosio and Donadio, NAICONS, Milano, Italy and is used by Bio S&T Inc. for PAC library construction. In contrast to previously developed *E. coli*–*Streptomyces* Artificial Chromosomes, pESAC13-A contains an *oriT* site that allows transfer into *Streptomyces* by conjugation and confers apramycin resistance in both *E. coli* and *Streptomyces*. pESAC13-A vector DNA was digested with *Bam*HI, dephosphorylated and purified using standard procedures. *S. koyangensis* SCSIO 5802 cells was embedded in 2 mL 2% low-melting-point agarose plugs, which was then treated with proteinase K at 50 °C and stored in 0.5 M EDTA at 4 °C. Plugs were partially digested with *Bam*HI at 37 °C and the reactions were stopped by the addition of 1/10 volume of 0.5 M EDTA (pH 8.0). For each case, partially digested high-molecular-weight DNAs were separated by two rounds of pulsed field gel electrophoresis (PFGE) with different ramped pulse times. The DNA fragments 100–300 kb were eluted from the gel by PFGE with a constant pulse time. The eluted DNA fragments were dialyzed against 1× TE (10 mM Tris–HCl, 1 mM EDTA, pH 8.0) prior to ligation. Partially-digested size-selected DNA fragments were ligated to the *Bam*HI-digested and dephosphorylated pESAC13-A vector. The ligation mix was transformed into *E. coli* DH10β by electroporation. Three sets of primers, including the primers for *abmB1*, *orf(*−*2)*, and *orf(*+*3)*, were designed to screen the library for the desired clone that contains all the biosynthetic genes.

The PAC clone 4-3F, which tested positive for all three PCR probes, was selected for heterologous expression. The DH10β *E. coli* strain containing 4-3F was used in a triparental mating with the non-methylating *E. coli* strain ET12567 containing the driver plasmid pUB307 and the heterologous host *S. coelicolor* M1152. For the *E. coli* strains DH10β/4-3F and ET12567/pUB307, they were grown to an OD_600_ of 0.4 in 10 mL LB; the cells were pelleted by centrifugation, washed in LB, pelleted again and finally resuspended in a volume of 200 μL LB. For the *S. coelicolor* M1152, the required number of spores (10^8^) were added in 400 μL TSB, then incubated at 50 °C for 10 min and continuously incubated at 28 °C for 5–6 h on rotary shakers (200 rpm) to activate germination. The germinated spores of *S. coelicolor* M1152 were mixed with the two previously prepared *E. coli* strains, and the mixture was spread onto an MS agar plate supplemented with 10 mM MgCl_2_. After incubation at 28 °C for 18 h, the plate was covered with 950 μL sterile deionized water containing 30 μL trimethoprim (TMP, 50 mg/mL) and 50 μL Apr (50 mg/mL). Finally, the plates were incubated for a further 4–5 days at 28 °C until exconjugants appeared. The resulting exconjugants were tested by PCR using two sets of primers, primers for *orf(*−*2)* and *orf(*+*3)*, to confirm that exconjugants contained the entire *abm* gene cluster.

### Overexpression of *abmI* or *abmH* in wild-type producer strain

The coding regions of *abmI* and *abmH* were amplified by PCR using the genomic DNA of *S. koyangensis* SCSIO 5802 wild-type strain and the primers listed in Additional file 1: Table S3. Each of the PCR products was digested with *Nde*I/*Spe*I then cloned into the same digested sites of a pSET152-derived expression plasmid, pL646 [17], under the control of a strong constitutive promoter ermE*p. The recombinant plasmids pL646-abmI and pL646-abmH were transformed into *E. coli* ET12567/pUZ8002, and then individually transferred into wild-type *S. koyangensis* SCSIO 5802 by conjugation; the exconjugants were selected on the basis of phenotypes showing apramycin resistance and then confirmed by PCR to give the overexpression strains 5802::*abmI* and 5802::*abmH*.

### Metabolite analyses of wild-type *S. koyangensis* SCSIO 5802 and related derivative strains

The wild-type *S. koyangensis* SCSIO 5802 and relevant gene-inactivated mutants were first grown on A1 medium agar [10] at 28 °C for 5 days to achieve sporulation. For the *abmA1*–*A5* mutant strains, a portion of mycelium and spores (1 cm^2^) for each strain was added to 250 mL flasks containing 50 mL of RA medium [10] with supplemental 1% XAD-16 resin. Fermentations were then carried out at 28 °C on rotary shakers (200 rpm) for 8 days. After fermentation, each fermentation culture was centrifuged to yield the supernatant and pellet. Pellets were washed with 30 mL MeOH three times, and, for each sample, the MeOH extracts were combined, and the MeOH solvent was removed under reduced pressure to afford an oily residue. Residues were each dissolved into 1 mL MeOH and centrifuged at 13,000*g* for 10 min; supernatants were subjected to HPLC–UV analyses, each of which was performed using an Agilent Technologies 1260 Infinity system using a Phenomenex ODS column (150 × 4.6 mm, 5 μm), eluting with a linear gradient of 5 to 65% solvent B (solvent B: CH_3_CN + 0.1% trifluoroacetic acid (TFA); solvent A: H_2_O + 0.1% TFA) over 20 min, followed by 65% to 100% solvent B in 2 min, and then 100% solvent B for 5 min, at a flow rate of 1 mL/min. In all cases chromatograms employed UV detection at 254 nm. To characterize compounds **5** and **6** that accumulated in the Δ*abmA4* and Δ*abmA5* mutants, the metabolites of these two mutants were further subjected to mass analysis. MS analyses for **5** and **6** were carried out on a Bruker MaXis quardrupole-time-of-flight mass spectrometer equipped with an electrospray iron source (operated in the positive ion mode) fitted with a Phenomenex ODS column (150 × 4.6 mm, 5 μm) using a gradient of solvent B as described above for HPLC–UV analyses.

For other gene-inactivated mutants, the recombinant heterologous *abm* expression strains and the *abmI*/*abmH* overexpression strains, a portion of mycelium and spores (1 cm^2^) of each strain was inoculated into 250-mL flasks containing 50 mL of RA medium without supplemental of 1% XAD-16 resin. Fermentations were then carried out at 28 °C on rotary shakers (200 rpm) for 8 days. After fermentations, each culture broth was extracted with two volumes of butanone (1 × 100 mL), and solvents were then removed under reduced pressure to afford oily residue. Each residue was dissolved into 1 mL MeOH and centrifuged at 13,000*g* for 10 min; the supernatant was subjected to HPLC–UV analyses according to the methods described above except that the mobile phases did not contain 0.1% TFA.

To quantify the production of abyssomicin 2 (**3**) from wild-type and *abmI*/*abmH* overexpression strains, a standard curve was first generated using an analytically pure sample and HPLC–UV analyses (Additional file 1: Figure S11). Using a detection wavelength of 254 nm and correspondingly integrated signals (relative to control injections) the concentrations of abyssomicin 2 (**3**) were determined for the wild-type and *abmI*/*abmH* overexpressing strains.

## Results and discussion

### Identification of the neoabyssomicin/abyssomicin BGC and determination of the gene cluster boundaries

**Fig. 2:**
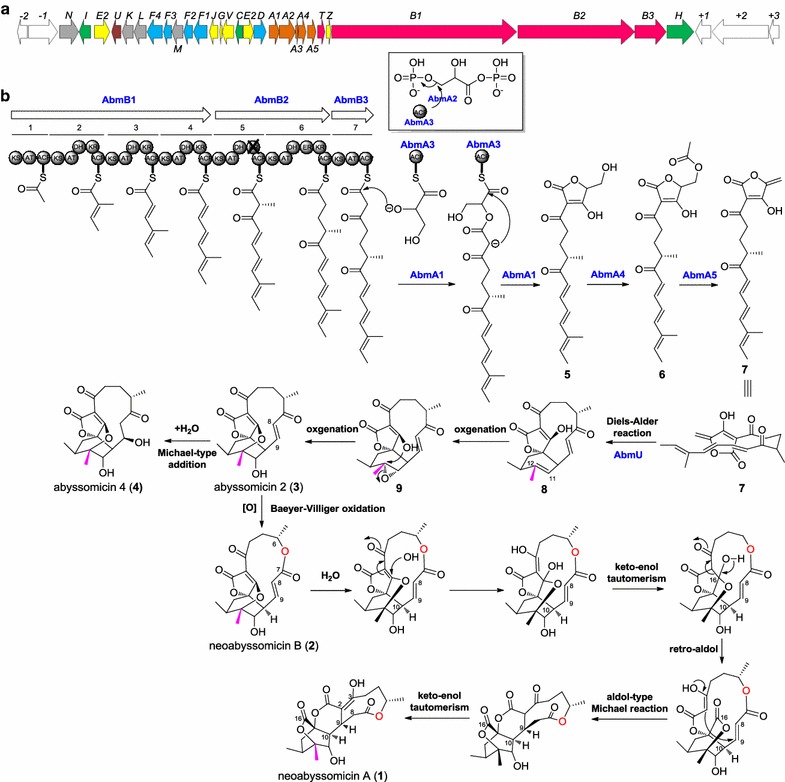
Organization of the *abm* BGC (**a**) and proposed biosynthetic pathway (**b**) for neoabyssomicins/abyssomicins in *S. koyangensis* SCSIO 5802

To locate the full BGC coding for neoabyssomicin/abyssomicin biosynthesis, the complete genome for *S. koyangensis* SCSIO 5802 was sequenced and characterized; the genome was found to contain 7,102,961 bp of DNA sequence. Bioinformatics analysis of the *S. koyangensis* SCSIO 5802 genome data using online antiSMASH software identified a polyketide synthase (PKS) I gene cluster, containing 28 ORFs and spanning 62.9 kb of contiguous genomic DNA, as the putative neoabyssomicin/abyssomicin BGC (termed herein *abm*). Notably, about 75% of the newly identified ORFs showed similarity with homologues from the previously known *atrop*-abyssomicin C BGC (*aby*) from *V. maris* AB-18-032 [11] (Table 1 and Fig. 2a). The *abm* cluster contained three consecutive PKS I genes (*abmB1*–*B3*, homologues of *abyB1*–*B3* in *aby*) coding for assembly of the neoabyssomicin/abyssomicin polyketide backbone; located two ORFs upstream of the PKS I genes, there are five consecutive genes (*abmA1*–*A5*) homologous to *abyA1*–*A5* in *aby* and proposed to code for tetronate biosynthesis (Additional file 1: Figure S1). Additionally, the genes associated with biosynthetic Diels–Alder chemistry (*abmU*), oxygenation (*abmV*), as well as genes having to do with transport (*abmD*, *abmF1*–*F4*) and regulatory (*abmI* and *abmH*) functions, were also found in the *abm* BGC and showed homologies to corresponding counterparts in the *atrop*-abyssomicin C pathway (Table 1). Notably, there are another seven genes (*abmK*, *abmL*, *abmM*, *abmN*, *abmJ*, *abmG* and *abmE2*) in *abm* with no apparent homologous counterparts in the *aby* cluster (Table 1).

**Table 1:** Deduced functions of ORFs in *abm* BGC from *S. koyangensis* SCSIO 5802

ORF	Size^a^	Proposed function	Closest homolog, origin (protein ID); ID/SI (%)	*aby* homolog	*abs* homolog
*orf(*−*2)*	223	Potassium uptake protein	TrkA, *Streptomyces coelicolor* A3(2) (Q53949.2); 88/96	–	–
*orf(*−*1)*	687	Amino acid permease	PlaP, *Escherichia coli* O157:H7 (P0AA48.1); 21/39	–	–
*abmN*	445	rRNA (Uracil-5-)-methyltransferase	RlmD, *Acinetobacter baumannii* AB307-0294 (B7H018.1); 30/48	–	–
*abmI*	268	Transcriptional activator, SARP family	DnrI, *Streptomyces peucetius* (P25047.1); 35/50	*abyI*	–
*AbmE2*	356	Luciferase-like monooxygenase, α-subunit	LuxA, *Photorhabdus luminescens* (P23146.1); 24/42	–	–
*abmU*	218	Diels–Alderase	YD repeat-containing protein, *Streptomyces regensis* (KMS84434.1); 41/52	*abyU*	*absU*
*abmK*	256	4′-Phosphopantetheinyl transferase superfamily (PPTase)	Npt, *Nocardia iowensis* (A1YCA5.1); 42/54	–	–
*abmL*	281	Metallophosphoesterase	GsiA, *Salmonella enterica* (Q57RB2.2); 46/60	–	–
*abmF4*	560	ABC transporter system ATP-binding protein	OppD, *Lactococcus lactis* (AIS04392.1); 42/73 or OppF, *Lactococcus lactis* (ABA47382.1); 43/74	*abyF4*	*absF4*
*abmF3*	298	ABC transporter system substrate-binding protein dependent permease	OppC, *Lactococcus lactis* (ABA47380.1); 27/63	*abyF3*	*absF3*
*abmM*	413	Amidohydrolase	Mb2939c, *Mycobacterium bovis* AF2122/97 (P68916.1); 28/37	–	–
*abmF2*	313	ABC transporter system permease	OppB, *Lactococcus lactis* (ABA47381.1); 27/58	*abyF2*	*absF2*
*abmF1*	546	ABC transport system substrate-binding protein	OppA, *Lactococcus lactis* (AAO63469.1); 20/52	*abyF1*	*absF1*
*abmJ*	331	Aldo/keto reductase	OsI_15387, *Oryza sativa* Indica Group (A2XRZ0.1); 50/68	–	*absJ*
*abmG*	77	Ferredoxin	Fd-1, *Streptomyces griseolus* (P18324.3); 58/75	–	*absG1*
*abmV*	405	Cytochrome P450	Vitamin D3 dihydroxylase, *Streptomyces griseolus* (P18326.2); 55/70	*abyV*	*absV*
*abmC*	257	TetR regulatory protein	Mce3R, *Mycobacterium tuberculosis* H37Rv (P95251.2); 33/48	*abyC*	*absC2*
*AbmE1*	353	Luciferase-like monooxygenase, β-subunit	LuxB, *Photorhabdus luminescens* (P19840.1); 20/41	*abyE*	–
*abmD*	487	Major facilitator superfamily of transporter	EmrB, *Mycobacterium tuberculosis* CDC1551 (P9WG88.1); 34/56	*abyD*	*absD*
*abmA1*	343	Ketoacyl-*S*-ACP synthase	ChlM, *Streptomyces antibioticus* (AAZ77702.1); 61/74	*abyA1*	*absA1*
*abmA2*	628	Glyceryl-*S*-ACP synthase	ChlD, *Streptomyces antibioticus* (AAZ77703.1); 61/70	*abyA2*	*absA2*
*abmA3*	75	Acyl carrier protein	ChlD2, *Streptomyces antibioticus* (AAZ77704.1); 56/73	*abyA3*	*absA3*
*abmA4*	280	2-Oxoacid dehydrogenase multienzymes acyltransferase E2 component	ChlD3, *Streptomyces antibioticus* (AAZ77705.1); 65/76	*abyA4*	*absA4*
*abmA5*	373	α/β hydrolase fold protein	ChlD4, *Streptomyces antibioticus* (AAZ77706.1); 51/64	*abmA5*	*absA5*
*abmT*	274	Type II thioesterase	PikA5, *Streptomyces venezuelae* (Q9ZGI1.1); 32/45	*abyT*	–
*abmZ*	178	NADPH-dependent flavin reductase	HsaB, *Rhodococcus jostii* RHA1 (Q0S808.1); 39/57	*abyZ*	*absH1*
*abmB1*	6540	PKS I (module 1: KS, ATa, ACP; module 2: KS, ATp, DH, KR, ACP; module 3: KS, ATa, DH, KR, ACP; module 4: KS, ATa, DH, KR, ACP)	PikA1, *Streptomyces venezuelae* (Q9ZGI5.1); 54/65	*abyB1*	*absB1*
*abmB2*	4054	PKS I (module 5: KS, ATp, DH, KR, ACP; module 6: KS, ATa, DH, ER, KR, ACP)	PikA2, *Streptomyces venezuelae* (Q9ZGI4.1); 49/59	*abyB2*	*absB2*
*abmB3*	1040	PKS I (module 7: KS, ATa, ACP)	PikA1, *Streptomyces venezuelae* (Q9ZGI5.1); 54/64	*abyB3*	*absB3*
*abmH*	942	LuxR family transcriptional regulator	NreC, *Staphylococcus carnosus* subsp. carnosus TM300 (Q7WZY4.1); 48/67	*abyH*	–
*orf(*+*1)*	530	Hypothetical protein	Hypothetical protein*; Actinospica acidiphila* (WP_033273634.1); 49/58	–	–
*orf(*+*2)*	1939	Hypothetical protein	Hypothetical protein, *Actinospica acidiphila* (WP_033273633.1); 56/67	–	–
*orf(*+*3)*	331	Alcohol dehydrogenase-like protein	TDH, *Agrobacterium radiobacter* K84 (B9J738.1); 32/53	–	–

^a^Size in units of amino acids (aa); ID/SI: identity/similarity; *aby*: the BGC of *atrop*-abyssomicin C from *Verrucosispora* sp. AB-18032; *abs*: the putative BGC of abyssomicins M–X from *Streptomyces* sp. LC-6

**Fig. 3:**
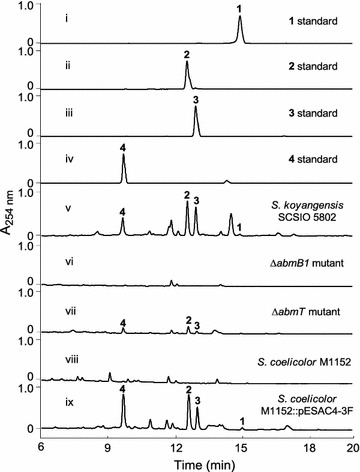
HPLC analyses of fermentation extracts. i–iv: authentic standards of compound **1**–**4**; v: wild-type *S. koyangensis* SCSIO 5802; vi: Δ*abmB1* mutant; vii: Δ*abmT* mutant; viii: negative control of the host strain *S. coelicolor* M1152; ix: *S. coelicolor* M1152 bearing the 4-3F BAC clone, which contains the *abm* cluster

To confirm the validity of the identified *abm*, we inactivated PKS I in *abmB1* within the *S. koyangensis* producer using established λ-RED-mediated PCR-targeting mutagenesis methods. As expected, the production of neoabyssomicins/abyssomicins was completely abolished in the Δ*abmB1* mutant strain (Fig. 3, trace vi), thus demonstrating that *abm* is indeed responsible for neoabyssomicin/abyssomicin biosynthesis.

BLASTP analyses and comparisons with the known *atrop*-abyssomicin C BGC suggested that *abmI* and *abmH* may represent upstream and downstream boundaries of *abm*, respectively. Upstream of *abm*, two genes, *orf(*−*1)* and *orf(*−*2)* were found to encode an amino acid permease and a potassium uptake protein, respectively, with no homologous counterparts in the *aby* BGC. These two types of proteins are frequently involved in primary metabolic processes. Accordingly, *orf(*−*1)* and *orf(*−*2)* appeared to have no involvement in the biosynthesis of neoabyssomicins/abyssomicins. Downstream of the *abm* cluster, *orf(*+*1)* and *orf(*+*2)*, encoding two hypothetical proteins; and *orf(*+*3)*, encoding an alcohol dehydrogenase-like protein and also having no homologous counterparts in the *aby* cluster, were also ruled out as players in neoabyssomicin/abyssomicin biosynthesis. Confirming these assertions, gene inactivations for *orf(*−*1)*, *orf(*+*1)*, *orf(*+*2)* and *orf(*+*3)* revealed that none of these genes (and their putative products) play a role in neoabyssomicin/abyssomicin biosynthesis (Additional file 1: Figure S2). Correspondingly, these efforts helped to delineate the boundaries of the *abm* cluster.

### Biosynthesis and assembly of the polyketide backbone

There are three consecutive type I PKS genes in the *abm* cluster encoding a total of seven PKS modules for assembly of the neoabyssomicin/abyssomicin polyketide backbone. The first gene, *abmB1*, consists of four modules. The first module consists of the minimal set of ketosynthase (KS), acyltransferase (AT) and acyl carrier protein (ACP) domains. The active site Cys (for transthioesterification) of the KS in this module is replaced by Gln (Additional file 1: Figure S3); consequently, this KS is a “KS_Q_” domain often found in loading modules of PKS systems [18]. This KS_Q_ acts as a loading module for formation of the acetate starter unit by catalyzing decarboxylation of an ACP-tethered malonate. In the subsequent three modules of *abmB1*, in addition to the minimal set of KS, AT and ACP, a dehydratase (DH) domain and a ketoreductase (KR) domain are also present, arranging collinearly with their functions in the biosynthetic assembly line. Module 5 of *abmB2* has the same arrangement as module 4 although its KR domain is believed to be inactive on the basis of sequence analyses indicating that it lacks both the conserved NADPH binding motif and the Lys and Tyr of the active site triad required of reductase activity [19]; these active site residues are replaced by Glu and Phe, respectively (Additional file 1: Figure S4). Whether the DH domain of module 5 (Additional file 1: Figure S5) is active or not is unclear since the requisite hydroxy group of a putative DH substrate is absent due to crippling of the KR domain (Additional file 1: Figure S4). In contrast to module 5, module 6 of *abmB2* contains an additional enoylreductase (ER) domain. Module 7 of *abmB3* consists of a minimal set of KS, AT and ACP that adds an additional acetate unit. AbmB1-AT_2 and AbmB2-AT_5 contain the substrate specificity code (YASH) [20] (Additional file 1: Figure S6), characteristic of propionate incorporation. The remainder of the five AT domains contain the substrate specificity code (HAFH) [20] (Additional file 1: Figure S6), indicative of malonyl-CoA specificity; this observation is consistent with the structure of neoabyssomicins/abyssomicins.

Discrete type II TEs have been reported to play a role in editing stalled polyketide chains [21]. The gene *abmT* encodes a discrete type II thioesterase (TE) and multiple sequence alignments of AbmT with the typical type II TEs revealed that it contains the conserved motif (GHSXG) (Additional file 1: Figure S7) [22, 23]. Therefore, AbmT probably plays an editing role in the biosynthesis of neoabyssomicins/abyssomicins by hydrolyzing misincorporated monomers. Notably, *abmT* inactivation was found to decrease neoabyssomicin/abyssomicin titers relative to the wild-type producer consistent with its predicted editing function (Fig. 3, trace vii).

### Formation of the tetronate moiety and subsequent post-tailoring steps

Within the *abm* cluster *abmA1*–*A5* are homologous to a set of five highly conserved genes unique to tetronate biosynthesis (Additional file 1: Figure S1). Early biochemical studies unveiled a common pathway relevant to most (if not all) tetronate-containing natural products. The pathway is composed of the following steps: (i) transfer of a glycerol moiety from d-1,3-biphosphoglycerate to a discrete acyl carrier protein (ACP) (e.g., Tmn7a in tetronomycin biosynthesis [24], a homologue of AbmA3) as catalyzed by a glyceryl-*S*-ACP synthase (e.g., RkE in RK-682 biosynthesis [25], a homologue of AbmA2), leading to glyceryl-*S*-ACP; (ii) binding of the glyceryl-*S*-ACP to the nascent polyketide chain and detachment of the polyketide from the PKS, generating the linear hydroxymethyl tetronate ring as catalyzed by a ketoacyl-*S*-ACP synthase (e.g., RkD in RK-682 biosynthesis [26], a homologue of AbmA1); (iii) exomethylene installation via an acylation-elimination process accomplished by an acyltransferase E2 component of 2-oxoacid dehydrogenase multienzymes (e.g., Agg4 in agglomerin biosynthesis [27] and QmnD3 in quartromicin biosynthesis [28], homologues of AbmA4) and an α/β hydrolase fold protein (e.g., Agg5 in agglomerin biosynthesis [27] and QmnD4 in quartromicin biosynthesis [28], homologues of AbmA5). This sequence of steps, executed by the products of *abmA1*–*A5*, likely accounts for tetronate generation in the neoabyssomicins/abyssomicins in a fashion highly similar to other tetronates such as agglomerin, quartromicin and others (Fig. 2b for a proposed biosynthetic pathway).

**Fig. 4:**
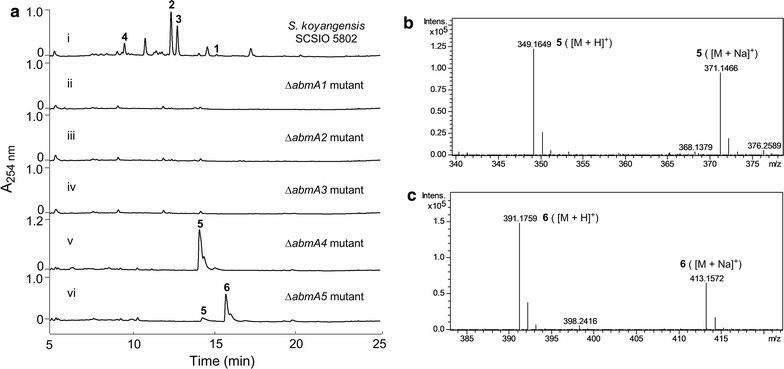
Analyses of the fermentation extracts of *abmA1*–*A5* inactivated mutants: **a** HPLC–UV analyses of the fermentation extracts of *abmA1*–*A5* inactivated mutants. **b** HR–ESI–MS spectra of peak **5** of the fermentation extract of Δ*abmA4* mutant. **c** HR–ESI–MS spectra of peak **6** of the fermentation extract of Δ*abmA5* mutant

To validate our hypotheses regarding tetronate installation and to define the roles of *abmA1*–*A5*, each was specifically inactivated to afford double-crossover mutants for each gene. Inactivation of *abmA1*, *abmA2 and abmA3* completely abolished neoabyssomicin/abyssomicin production, confirming their involvement in neoabyssomicin/abyssomicin biosynthesis (Fig. 4a, traces ii–iv). HPLC–UV analyses of the fermentation extract for the Δ*abmA4* mutant revealed its failure to produce neoabyssomicins/abyssomicins (**1**–**4**) (Fig. 4a, trace v). However, the Δ*abmA4* mutant accumulated a new compound whose molecular mass, as determined by HR–ESI–MS ([M + H]^+^, calculated: 349.1606, found: 349.1649) (Fig. 4b), exactly matched that predicted for linear hydroxy-tetronate intermediate **5** (Fig. 2b). The Δ*abmA5* mutant, on HPLC–UV analysis of its fermentation extract, also failed to produce **1**–**4** but accumulated minor amounts of **5** (Fig. 4a, trace vi). In addition, the Δ*abmA5* mutant, generated a major metabolite with longer HPLC elution time than **5** (Fig. 4a, trace vi) and whose molecular mass, on the basis of HR–ESI–MS ([M + H]^+^, calculated: 391.1712, found: 391.1759) (Fig. 4c), exactly matched that predicted for the acetyl-tetronate intermediate **6** (Fig. 2b). Consequently, it is clear on the basis of these inactivation studies that *abmA1*–*A5* all play vital roles in **1**–**4** biosynthesis and that, more precisely, *abmA4* and *abmA5* are involved in tetronate exomethylene installation leading to **7**; *abmA4* codes for the OH activating acetyltransferase and the *abmA5* product catalyzes elimination to generate the exomethylene moiety.

We hypothesized early on that intermediate **7** may readily undergo an intermolecular [4 + 2] cycloaddition (Diels–Alder) reaction between the newly installed exomethylene group and the terminal conjugated diene to afford polycyclic species **8** uniquely characterized by its spirotetronate skeleton. AbmU, showing sequence similarity (37% identity) to the Diels–Alderase, AbyU [12], in the *atrop*-abyssomicin C biosynthetic pathway, was proposed to catalyze this intramolecular spirocyclization. The path from polycycle **8** to neoabyssomicins/abyssomicins (**1**–**4**) dictates the involvement of several tailoring enzymes. We envision that **8** undergoes epoxidation of the cyclohexene C11–C12 double bond affording epoxide **9**, which then reacts via intramolecular ring-opening with the sole OH moiety. Epoxide opening via nucleophilic attack by the tetronate OH affords abyssomicin 2 (**3**). Hydration of **3** via Michael-type addition to the C8–C9 olefin can be readily envisioned as a means of generating abyssomicin 4 (**4**). Structurally, neoabyssomicins A and B (**1**, **2**) constitute a new subtype (type II_C_) of abyssomicins, featuring an inserted oxygen atom within the polyketide chain, and we proposed that a unique biosynthetic Baeyer–Villiger reaction represents access to this new class of abyssomicins. We envision that **3** might also undergo Baeyer–Villiger oxidation to yield neoabyssomicin B (**2**). Notably, the new scaffold neoabyssomicin A (**1**) may well be derived from **2** through a sequence of: (i) C-16 hydrolysis, (ii) keto-enol tautomerism, (iii) a retro-aldol reaction to establish the C-16 containing lactone, (iv) establishment of the C-2 to C-9 linkage via an aldol-type Michael reaction, and finally, (v) a simple keto-enol tautomerization (Fig. 2b). Within the *abm* cluster, *abmV*, *abmM*, *abmJ*, *abmG*, *abmE1* and *abmE2*, are all predicted to encode enzymes related to oxidation or reduction (Table 1) and are therefore excellent tailoring enzyme candidates for the **8** → **1**–**4** progression. However, although their involvement in these conversions is highly likely, the precise details for how these gene products carry out their relevant chemistries remains to be determined.

### Export and import systems for neoabyssomicin/abyssomicin biosynthesis

**Fig. 5:**
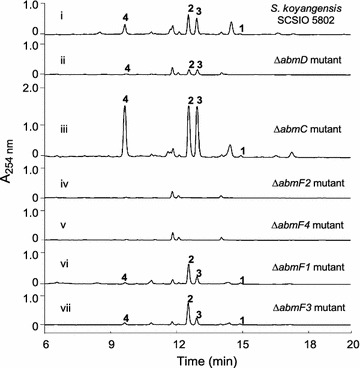
HPLC analyses of fermentation extracts. i: wild-type *S. koyangensis* SCSIO 5802; ii: Δ*abmD* mutant; iii: Δ*abmC* mutant; iv: Δ*abmF2* mutant; v: Δ*abmF4* mutant; vi: Δ*abmF1* mutant; vii: Δ*abmF3* mutant

The *abmD orf* is predicted to encode a major facilitator superfamily (MFS) protein. MFS family proteins possess 12 or 14 transmembrane segments (TMS) and are capable of transporting small molecules in response to changes in chemiosmotic gradients [29]. Detailed sequence analysis of AbmD revealed that it belongs to the 14-TMS subfamily of the MFS (Additional file 1: Figure S8). In *Streptomyces*, members of the 14-TMS subfamily are usually involved in antibiotic secretion from the producer strain thus endowing the microbial producer with a self-defense mechanism [30, 31]. Since neoabyssomicins/abyssomicins show antibacterial activity, we posited that AbmD might be involved in cellular export of neoabyssomicin/abyssomicin; such a transport system is likely needed to protect the bacterial cell from the effects of these secondary metabolites. Consistent with a vital role in export, inactivation of *abmD* dramatically decreased neoabyssomicin/abyssomicin production (Fig. 5, trace ii). Supportive of this assertion, we found that *abmC* encodes a TetR-like regulator and is divergently transcribed from a bicistronic transcript encoding for AbmE1 and AbmD. Notably, *abmC* inactivation correlated to significantly increased neoabyssomicin/abyssomicin production relative to the wild-type producer (Fig. 5, trace iii). These data illustrate an important relationship between *abmD* and *abmC* (and each one’s putative roles) that is commonly observed in other systems and that emphasizes the importance of *abmD* in neoabyssomicin/abyssomicin production; it is well known that the expression of drug transporters in the 14-TMS subfamily are frequently repressed by TetR-family regulators, which are transcribed divergently from adjacent target genes [30–32]. Thus, the results of *abmD* inactivation studies, combined with the assignment of AbmC function and association with *abmD* expression make clear that *abmD* codes for a putative neoabyssomicin/abyssomicin transporter/resistance protein in *S. koyangensis* SCSIO 5802.

Upstream of *abmD* are *abmF1*–*F4*, which encode an ABC transporter system. The ABC transporters are a large family of membrane-associated export and import systems [33, 34]. Members of this family share a common four-domain organization: two transmembrane domains (TMDs) that span the membrane several times in α-helical conformation and two membrane associated nucleotide-binding domains (NBDs) that bind ATP and couple its hydrolysis to the transport process [34]. Most prokaryotic ABC transporters are encoded as separate TMD and NBD polypeptides, which form half transporters that dimerize to generate a fully functional transporter [34]. In addition, bacterial ABC import systems contain an additional extracellular substrate-binding protein (SBP) that captures the substrate and delivers it to the translocator. For instance, the oligopeptide ABC import system (Opp) from *Lactococcus lactis* [35], is a five-component ABC transporter composed of a membrane-anchored SBP, OppA, two transmembrane proteins, OppB and OppC, and two ATP-binding domains, OppD and OppF. On the basis of these established genes, their products and the mechanisms by which they function in concert we can glean significant insight into the *abm* cluster components with parallel roles. According to the sequence data, AbmF1, AbmF2 and AbmF3 are, respectively, the OppA (identity/similarity: 20.2%/52.8%), OppB (identity/similarity: 27.7%/58.3%) and OppC (identity/similarity: 27.3%/63.1%) homologues of the Opp system (Table 1). Interestingly, OppD and OppF homologues in the AbmF1–F4 system are fused together and encoded as one polypeptide (by *abmF4*); AbmF4 consists of two NBD domains. In total, and based on comparisons to known import systems, it would appear that *abmF1*–*F4* likely serves the role of an importer system for neoabyssomicin/abyssomicin biosynthesis within its microbial producer. The identity of the putative “substrate” imported by AbmF1–F4 is not yet established but clearly warrants further investigation.

Individual deletion of *abmF2* (encoding one of the two transmembrane proteins) and *abmF4* (encoding an ATP-binding protein) both totally abolished the neoabyssomicin/abyssomicin production (Fig. 5, traces iv, v); individual deletions of *abmF1* (encoding the extracellular substrate-binding protein) and *abmF3* (encoding another transmembrane protein) dramatically reduced titers of abyssomicin 2 (**3**) and abyssomicin 4 (**4**) to less than ~ 10% of the wild-type level, although the yields of neoabyssomicin B (**2**) produced by each of these two mutant strains was on par with that of the wild-type strain (Fig. 5, traces vi, vii). These data further support the notion that *abmF1*–*F4* are likely involved in the cellular import of a compound required for neoabyssomicin/abyssomicin production.

### AbmI and AbmH are two positive regulators governing neoabyssomicin/abyssomicin production

Within the *abm* cluster *abmI* and *abmH*, encode homologues of known regulators of secondary metabolism. The *abmI* gene, present in the far upstream (left) region of the cluster, encodes a protein with significant similarity to pathway specific *Streptomyces* antibiotic regulatory protein (SARP) transcriptional activators, such as ActII-ORF4 [36], RedD [37] and DnrI [38]. Sequence alignments of AbmI with these well-characterized SARP regulators revealed that they all contain a conserved N-terminal OmpR-like DNA-binding domain and a C-terminal bacterial transcriptional activation domain (BTAD) (Additional file 1: Figure S9), indicating that AbmI might be a positive regulator of neoabyssomicin/abyssomicin biosynthesis. Located within the other far extreme side (downstream) of the *abm* cluster was found *abmH* encoding a transcriptional regulator of the LuxR family. In Gram-negative bacteria, LuxR regulators, composed of an N-terminal autoinducer binding domain and a C-terminal helix-turn-helix (HTH) DNA-binding domain, can be activators or repressors, which, acting through a quorum sensing mechanism, induce transcription when a certain cell density is reached [39, 40]. In actinobacteria, LuxR regulators are usually identified as pathway specific transcriptional activators such as AveR [41], PikD [42], and GdmR1 [43]; these systems are characterized by an N-terminal nucleotide triphosphate (NTP)-binding domain represented by Walker A motif (GxxxxGK[T/S]) and Walker B motif (hhhhD) (where h is a hydrophobic residue), as well as a C terminal HTH LuxR-type DNA-binding domain. Bioinformatics analyses revealed that AbmH showed sequence and domain organizations similar to those of the well-studied AveR, PikD, and GdmR1 regulators (Additional file 1: Figure S10). Furthermore, AbmH appears to lack any obvious autoinducer binding domain (Additional file 1: Figure S10) suggesting AbmH as a likely positive regulator of neoabyssomicin/abyssomicin production.

**Fig. 6:**
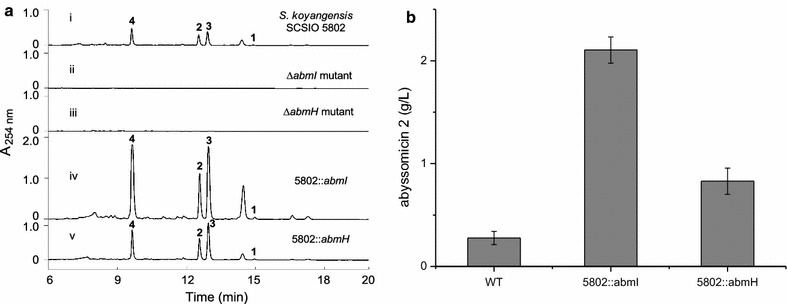
Individual overexpression of *abmI* and *abmH* enhances neoabyssomicin/abyssomicin production. **a** HPLC analyses of fermentation extracts. i: wild-type *S. koyangensis* SCSIO 5802; ii: Δ*abmI* mutant; iii: Δ*abmH* mutant; iv: *abmI* overexpression strain 5802::*abmI*; v: *abmH* overexpression strain 5802::*abmI*. 5 μL fermentation extract was subjected to HPLC analyses for each of the samples. **b** Abyssomicin 2 (**3**) production of wild-type and overexpression strains. WT: wild-type *S. koyangensis* SCSIO 5802. The values are mean ± SD from three different experiments

To determine the roles of *abmI* and *abmH* in **1**–**4** biosynthesis, both genes were independently inactivated according to standard methods to generate the Δa*bmI* and Δa*bmH* mutants, respectively. As expected, disruption of *abmI* and *abmH* completely abolished neoabyssomicin/abyssomicin production (Fig. 6a, traces ii, iii). These results demonstrated that *abmI* and *abmH* are both pivotal positive regulators of neoabyssomicin/abyssomicin biosynthesis.

### Heterologous expression of the *abm* BGC efficiently produces **1**–**4**

Based on the above informatics and genetic information, we hypothesized that all genes required for biosynthesis of all four neoabyssomicins/abyssomicins (**1**–**4**) are present in the identified *abm* cluster, although several putative tailoring genes/enzymes and mechanisms are not yet well-defined. To verify the ability of *abm*-housed genes to generate **1**–**4**, we sought to heterologously express the *abm* BGC in *S. coelicolor* M1152. A P1-derived artificial chromosome (PAC) library for the *S. koyangensis* SCSIO 5802 genome was constructed using an *E. coli*–*Streptomyces* artificial chromosome vector, pESAC-13-A (a derivative of pPAC-S1 [16]), which can be shuttled between *E. coli* and a suitable *Streptomyces* host. Three sets of primers, including the primers for *abm1*, *orf(*−*2)*, and *orf(*+*3)*, were used to screen ~ 2000 clones of the library for the one containing all the identified biosynthetic genes. A BAC clone, 4-3F, testing positive for all three PCR probes, was isolated and introduced into *S. coelicolor* M1152 by triparental intergeneric conjugation to generate M1152::pESAC4-3F, with the integrated *abm* gene cluster in its chromosome. HPLC–UV analyses of the fermentation extract of M1152::pESAC4-3F revealed that, indeed, this heterologous host produced **1**–**4** in yields rivaling the wild-type producer (Fig. 3, traces viii, ix); BAC clone 4-3F clearly housed the intact *abm* cluster containing all genes needed for wild-type levels of neoabyssomicin/abyssomicin production.

### Overexpression of *abmI* or *abmH* boosts neoabyssomicin/abyssomicin production

Since *abmI* and *abmH* both encode pathway-specific regulators, positively regulating the production of neoabyssomicin/abyssomicin, overexpression of *abmH* or *abmH* was envisioned to be a viable means of increasing neoabyssomicin/abyssomicin titers in *S. koyangensis* SCSIO 5802. Similarities to related systems [36–43] and the results indicated in Fig. 6 supported this logic. To evaluate this hypothesis each positive regulator *abmI* and *abmH* was independently cloned into a pSET152-derived expression plasmid, pL646 [17]; both *abmI* and *abmH* were situated under the control of constitutive ermE*p promoter. Each of the resulting plasmids was conjugated into wild-type *S. koyangensis* SCSIO 5802 to create 5802:*abmI* and 5802:*abmH* overexpression strains, respectively. As expected, the yields for **1**–**4** increased almost uniformly relative to the unmodified wild-type producer (Fig. 6a, traces iv, v). Only abyssomicin 2 (**3**), the major metabolite in the fermentation, was quantitated in further experiments. Production of **3** in 5802::*abmI* (2.1045 g/L) relative to the wild-type producer (0.2764 g/L) improved by 7.6-fold whereas the increase observed with 5802::*abmH* (0.8292 g/L) was approximately threefold relative to wild-type (Fig. 6b). These data clearly support the positive regulatory roles played by AbmI and AbmH in the synthesis of **1**–**4** while also providing a glimpse of how data presented herein might be employed in future combinatorial biosynthesis efforts to better exploit the unique structures and activities of the neoabyssomicins and abyssomicins.

## Conclusions

We have identified and characterized the neoabyssomicin/abyssomicin BGC in *S. koyangensis* SCSIO 5802 by carrying out whole genome sequencing, systematic gene disruptions and heterologous expression experiments; the *abm* BGC generates **1**–**4**. Informatics analyses and genetics data have enabled us to propose a plausible biosynthetic pathway leading to **1**–**4**. Central to this proposal is our demonstration that the tetronate moieties result from an enzymatically driven acetylation-elimination sequence. Beyond their basic construction, we employed gene inactivations and bioinformatics to unveil an export system by which the microbial producer avoids the detrimental effects of **1**–**4** intracellular production; a biosynthetically vital four-component import system was also uncovered. Finally, we have also revealed that *abmI* and *abmH* both encode proteins involved in positively regulating neoabyssomicin/abyssomicin biosynthesis; overexpression of either gene afforded significant enhancements in titers. Overall, the findings detailed here set the stage for dramatic improvements in the production, study, and potential clinical applications of the neoabyssomicin/abyssomicin scaffold. The working model for biosynthesis (and its regulation) of **1**–**4** enabled by these efforts is anticipated to significantly advance combinatorial biosynthetic initiatives to better exploit this unique family of natural products.

## Additional file

**Additional file 1: Table S1.** Bacteria used in this study. **Table S2.** Plasmids used in this study. **Table S3.** Primers used in this study. **Figure S1.** Chemical structures of tetronate-containing natural products and the unique set of five highly conserved genes responsible for tetronate biosynthesis. **Figure S2.** HPLC analyses of fermentation extracts of the inactivated mutants of boundary genes. **Figure S3.** Alignments of seven KS domains of AbmB1–B3. **Figure S4.** Alignments of five KR domains of AbmB1–B2. **Figure S5.** Alignments of five DH domains of AbmB1–B2. **Figure S6.** Alignments of five AT domains of AbmB1–B2. **Figure S7.** Alignments of AbmT with the typical type II TEs. **Figure S8.** The 14 transmembrane helices of AbmD. **Figure S9.** Alignments of AbmI with previously characterized SARP regulators. **Figure S10.** Alignments of AbmH with previously characterized LuxR-regulators. **Figure S11.** The quantitative HPLC standard curve for abyssomicin 2. **Figures S12–S30.** Disruption of 19 *abm*-related genes in wild-type *S. koyangensis* SCSIO 5802 via PCR-targeting.

## Abbreviations

MRSA: methicillin-resistant *Staphylococcus aureus*
BGC: biosynthetic gene cluster
ORFs: opening reading frames
HIV: human immunodeficiency virus
*aby*: the biosynthetic gene cluster of *atrop*-abyssomicin C
*abs*: the putative biosynthetic gene cluster of abyssomicins M–X
*abm*: the biosynthetic gene cluster of neoabyssomicins/abyssomicins
PKS: polyketide synthase
AT: acyltransferase
ACP: acyl carrier protein
DH: dehydratase
ER: enoylreductase
KR: ketoreductase
TE: thioesterase
MFS: major facilitator superfamily
TMS: transmembrane segments
TMDs: transmembrane domains
NBDs: nucleotide-binding domains
SBP: substrate-binding protein
Opp: oligopeptide ABC import system
SARP: *Streptomyces* antibiotic regulatory protein
HTH: helix-turn-helix
BTAD: bacterial transcriptional activation domain
