# Supplementary material for: Characterization and heterologous expression of the neoabyssomicin/abyssomicin biosynthetic gene cluster from Streptomyces koyangensis SCSIO 5802
Source: Microb Cell Fact. 2018 Feb 20;17:28. doi: 10.1186/s12934-018-0875-1 (PMC5819245; doi:10.1186/s12934-018-0875-1)
Supplement: Supplementary file 1 — Additional file 1: Table S1. Bacteria used in this study. Table S2. Plasmids used in this study. Table S3. Primers used in this study. Figure S1. Chemical structures of tetronate-containing natural products and the unique set of five highly conserved genes responsible for tetronate biosynthesis. Figure S2. HPLC analyses of fermentation extracts of the inactivated mutants of boundary genes. Figure S3. Alignments of seven KS domains of AbmB1–B3. Figure S4. Alignments of five KR domains of AbmB1–B2. Figure S5. Alignments of five DH domains of AbmB1–B2. Figure S6. Alignments of five AT domains of AbmB1–B2. Figure S7. Alignments of AbmT with the typical type II TEs. Figure S8. The 14 transmembrane helices of AbmD. Figure S9. Alignments of AbmI with previously characterized SARP regulators. Figure S10. Alignments of AbmH with previously characterized LuxR-regulators. Figure S11. The quantitative HPLC standard curve for abyssomicin 2. Figures S12–S30. Disruption of 19 abm-related genes in wild-type S. koyangensis SCSIO 5802 via PCR-targeting. [file 12934_2018_875_MOESM1_ESM.pdf]

Characterization and heterologous expression of the neoabyssomicin/abyssomicin biosynthetic gene cluster from *Streptomyces koyangensis* SCSIO 5802

Jiajia Tu[1, 2+], Siting Li[1, 3+], Jiang Chen[1,4], Yongxiang Song[1], Shaobin Fu[2], Jianhua Ju[1, 4] and Qinglian Li[1]*

[1]CAS Key Laboratory of Tropical Marine Bio-resources and Ecology, Guangdong Key Laboratory of Marine Materia Medica, RNAM Center for Marine Microbiology, South China Sea Institute of Oceanology, Chinese Academy of Sciences, 164 West Xingang Road, Guangzhou 510301, China.

[2]School of Pharmacy, Zunyi Medical University, 201 Dalian Road, Zunyi 563000, China.

[3]College of Bio and Marine Sciences, Shenzhen University, 3688 Nanhai Ave, Shenzhen 518060, China.

[4]University of Chinese Academy of Sciences, 19 Yuquan Road, Beijing 110039, China.

[+]Equal contributors

*To whom correspondence should be addressed. Tel. /Fax: +86-20-34066449; E-mail: liql@scsio.ac.cn.

Email addresses of all co-authors:

Jiajia Tu: 1152492875@qq.com; Siting Li: li_sting@qq.com; Jiang Chen: chenjluck@126.com; Yongxiang Song: songx@scsio.ac.cn; Shaobin Fu: 407683156@qq.com; Jianhua Ju: jju@scsio.ac.cn; Qinglian Li: liql@scsio.ac.cn

Table of Contents **Pages**

Entry	Pages
**Table S1.** Bacteria used in this study.	2-3
**Table S2.** Plasmids used in this study.	4
**Table S3.** Primers used in this study.	5-7
**Fig. S1** Chemical structures of tetronate-containing natural products and the unique set of five highly conserved genes responsible for tetronate biosynthesis.	8
**Fig. S2** HPLC analyses of fermentation extracts of the inactivated mutants of boundary genes.	9
**Fig. S3** Alignments of seven KS domains of AbmB1–B3.	10
**Fig. S4** Alignments of five KR domains of AbmB1–B2.	11
**Fig. S5** Alignments of five DH domains of AbmB1–B2.	12
**Fig. S6** Alignments of seven AT domains of AbmB1–B3.	13
**Fig. S7** Alignments of AbmT with the typical type II TEs.	14
**Fig. S8** The 14 transmembrane helices of AbmD.	15
**Fig. S9** Alignments of AbmI with previously characterized SARP regulators.	16
**Fig. S10** Alignments of AbmH with previously characterized LuxR-regulators.	17-18
**Fig. S11** The quantitative HPLC standard curve for abyssomicin 2 (**3**)	19
**Fig. S12–30** Disruption of 19 *abm*-related genes in wild-type *S. koyangensis* SCSIO 5802 *via* PCR-targeting.	20-29

Table S1. Bacteria used in this study.

Strains	Description	Source/ [Ref.]
**Streptomycetes**		
Streptomyces coelicolor M1152	Host strain for heterologous expression of the *abm* biosynthetic gene cluster	
Streptomyces koyangensis SCSIO 5802	Wild-type producer of neoabyssomicins/abyssomicins (**1**–**4**)	This work
Δ*orf(-1)*	*S.koyangensis* SCSIO 5802 with a 900 bp fragment of *orf(-2)* substituted by *aac(3)IV+oriT* cassette	This work
Δ*abmI*	*S.koyangensis* SCSIO 5802 with a 609 bp fragment of *abmI* substituted by *a aac(3)IV+oriT* cassette	This work
Δ*abmF4*	*S.koyangensis* SCSIO 5802 with a 1005 bp fragment of *abmF4* substituted by *aac(3)IV+oriT* cassette	This work
Δ*abmF3*	*S.koyangensis* SCSIO 5802 with a 837 bp fragment of *abmF3* substituted by *aac(3)IV+oriT* cassette	This work
Δ*abmF2*	*S.koyangensis* SCSIO 5802 with a 684 bp fragment of *abmF2* substituted by *aac(3)IV+oriT* cassette	This work
Δ*abmF1*	*S.koyangensis* SCSIO 5802 with a 1545 bp fragment of *abmF1* substituted by *aac(3)IV+oriT* cassette	This work
Δ*abmD*	*S.koyangensis* SCSIO 5802 with a 1173 bp fragment of *abmD* substituted by *aac(3)IV+oriT* cassette	This work
Δ*abmA1*	*S.koyangensis* SCSIO 5802 with a 624 bp fragment of *abmA1* substituted by *aac(3)IV+oriT* cassette	This work
Δ*abmA2*	*S.koyangensis* SCSIO 5802 with a 1629 bp fragment of *abmA2* substituted by *aac(3)IV+oriT* cassette	This work
Δ*abmA3*	*S.koyangensis* SCSIO 5802 with a 138 bp fragment of *abmA3* substituted by *aac(3)IV+oriT* cassette	This work
Δ*abmA4*	*S.koyangensis* SCSIO 5802 with a 507 bp fragment of *abmA4* substituted by *aac(3)IV+oriT* cassette	This work
Δ*abmA5*	*S.koyangensis* SCSIO 5802 with a 744 bp fragment of *abmA5* substituted by *aac(3)IV+oriT* cassette	This work
Δ*abmB1*	*S.koyangensis* SCSIO 5802 with a 2649 bp fragment of *abmB1 substituted by aac(3)IV+oriT* cassette	This work
Δ*abmH*	*S.koyangensis* SCSIO 5802 with a 2391 bp fragment of *abmH substituted by aac(3)IV+oriT* cassette	This work
Δ*orf(+1)*	*S.koyangensis* SCSIO 5802 with a 1095 bp fragment of *orf(+1)* substituted by *aac(3)IV+oriT* cassette	This work
Δ*orf(+2)*	*S.koyangensis* SCSIO 5802 with a 2100 bp fragment of *orf(+2)* substituted by *aac(3)IV+oriT* cassette	This work
Δ*orf(+3)*	*S.koyangensis* SCSIO 5802 with a 738 bp fragment of *orf(+3)* substituted by *aac(3)IV+oriT* cassette	This work
5802::*abmI*	*abmI* overexpressed strain, dereived from *Streptomyces koyangensis* SCSIO 5802	This work
5802::*abmH*	*abmI* overexpressed strain, dereived from *Streptomyces koyangensis* SCSIO 5802	This work
E.coli		
DH5α	Host strain for general clone	
BW25113/pIJ 790	K-12 derivative: *araBAD*, *rhaBAD*; host strain for Red/ET-mediated recombination	[1]
ET12567/pU Z8002	*dam*, *dcm*, *hsdM*, *hsdS*, *hsdR, cat*^{R}, *tet*^{R}; donor strain for conjugation between *E.coli* and *Streptomyces*	[2]

XL 1-Blue MR	Host strain for construction of genomic cosmid library	Stratagene

Table S2. Plasmids used in this study.

Plasmids	Description	Source/ [Ref.]
**Plasmids**		
pIJ773	*aac(3)IV* (AprR), *oriT*; used for amplifying the aac(3)IV-oriT gene cassette for gene inactivation	[3]
pL646	Derived from pSET152, containing *ermE**P promoter and the ribosome-binding site of the *tuf1* gene, used for overexpression of *abmI* and abmH gene	[4]
SuperCos1	Used for construction of genomic cosmid library	Stratagene
cosmid 9-7C	A cosmid which contains partial *abm* biosynthetic cluster	This work
cosmid 6-7F	A cosmid which contains partial *abm* biosynthetic cluster	This work
cosmid 21-9A	A cosmid which contains partial *abm* biosynthetic cluster	This work
pΔ*orf(-1)*	A 900 bp fragment in *orf(-1)* in cosmid 6-7F was substituted by the *aac(IV)+oriT* cassette	This work
pΔ*abmI*	A 609 bp fragment in *abmI* in cosmid 6-7F was substituted by the *aac(IV)+oriT* cassette	This work
pΔ*abmF4*	A 1005 bp fragment in *abmF4* in cosmid 6-7F was substituted by the *aac(IV)+oriT* cassette	This work
pΔ*abmF3*	A 837 bp fragment in *abmF3* in cosmid 6-7F was substituted by the *aac(IV)+oriT* cassette	This work
pΔ*abmF2*	A 684 bp fragment in *abmF2* in cosmid 9-7C was substituted by the *aac(IV)+oriT* cassette	This work
pΔ*abmF1*	A 1545 bp fragment in *abmF1* in cosmid 9-7C was substituted by the *aac(IV)+oriT* cassette	This work
pΔ*abmD*	A 1173 bp fragment in *abmD* in cosmid 9-7C B was substituted by the *aac(IV)+oriT* cassette	This work
pΔ*abmA1*	A 624 bp fragment in *abmA1* in cosmid 9-7C was substituted by the *aac(IV)+oriT* cassette	This work
pΔ*abmA2*	A 1629 bp fragment in *abmA2* in cosmid 9-7C was substituted by the *aac(IV)+oriT* cassette	This work
pΔ*abmA3*	A 138 bp fragment in *abmA3* in cosmid 9-7C was substituted by the *aac(IV)+oriT* cassette	This work
pΔ*abmA4*	A 507 bp fragment in *abmA4* in cosmid 9-7C was substituted by the *aac(IV)+oriT* cassette	This work
pΔ*abmA5*	A 744 bp fragment in *abmA5* in cosmid 9-7C was substituted by the *aac(IV)+oriT* cassette	This work
pΔ*abmB1*	A 2649 bp fragment in *abmB1* in cosmid 9-7C was substituted by the *aac(IV)+oriT* cassette	This work
pΔ*abmH*	A 2391 bp fragment in *abmH* in cosmid 21-9A was substituted by the *aac(IV)+oriT* cassette	This work
pΔ*orf(+1)*	A 1095 bp fragment in *orf(+1)* in cosmid 21-9A was substituted by the *aac(IV)+oriT* cassette	This work
pΔ*orf(+2)*	A 2100 bp fragment in *orf(+1)* in cosmid 21-9A was substituted by the *aac(IV)+oriT* cassette	This work
pΔ*orf(+3)*	A 738 bp fragment in *orf(+3)* in cosmid 21-9A was substituted by the *aac(IV)+oriT* cassette	This work

Table S3. Primers used in this study.

Name	Sequence (5'-3')	Purpose
screen- abmB1-F	GCTGCGGAGTTGGTGGAGGA	For screening the *S.koyangensis* SCSIO 5802 genomic library
screen- abmB1-R	ACTGCGGTGTCGAGGAGGGA	
screen- orf(-2)-F	GCGGAGTTCGAGATCAAGCC	For screening the *S.koyangensis* SCSIO 5802 genomic library
screen- orf((-2)-R	CTGGAGCACCGTCTGGGAGG	
screen- orf(+3)-F	TGGAGCAGCAGGGGCACA	For screening the *S.koyangensis* SCSIO 5802 genomic library
screen- orf(+3)-R	GCTGCTCGTCCTCCTCTACACC	
orf(-1)-Del-F	GTGCTGCTGATCCTCTCGCTGGCGGGAGCGTCGGCGTACATTCCGGG GATCCGTCGACC	For disrupting *orf(-1)*
orf(-1)-Del-R	GACCATGACGCCGGGGGTGAAGTGGAGGCGCCCCTTGAGTGTAGGC TGGAGCTGCTTC	
abmI-Del-F	GAAGAGGGCGTCGCCGGGGTCGAGGCCCAGTTCTTCGCGATTCCGG GGATCCGTCGACC	For disrupting *abmI*
abmI-Del-R	TTCCTCGACCTGGCGCGAGCCGCGAGCGGCGATCCGGCCTGTAGGC TGGAGCTGCTTC	
abmF3-Del-F	ctcccgcagtcgggggtcgacgacccggtagagcccgtcATTCCGGGGATCCGTCGACC	For disrupting *abmF3*
abmF3-Del-R	accgccctgtgggtgacacgccggctgctcctcgcggtcTGTAGGCTGGAGCTGCTTC	
abmF2-Del-F	tgtctcccgcagtcgggggtcgacgacccggtagagcccATTCCGGGGATCCGTCGACC	For disrupting *abmF2*
abmF2-Del-R	cgcacgaccgccctgtgggtgacacgccggctgctcctcTGTAGGCTGGAGCTGCTTC	
abmF1-Del-F	cccacccaggctcacgtcgtggaacagcggatacgcgtgATTCCGGGGATCCGTCGACC	For disrupting *abmF1*
abmF1-Del-R	tccccgtcccgggcgggcggcagagcgctccgcgccgcgTGTAGGCTGGAGCTGCTTC	
abmD-Del-F	gtcaacgtcggcatcgactccctgggcaaggagttcgacATTCCGGGGATCCGTCGACC	For disrupting *abmD*
abmD-Del-R	gatgacggccgcgcccacgacccaccagaaggcgtggtgTGTAGGCTGGAGCTGCTTC	
abmA1-Del-F	atggacgccctgtgggagcagtgggtcgacgccttcatcATTCCGGGGATCCGTCGACC	For disrupting *abmA1*
abmA1-Del-R	cagggcctgttcgagctggaagaagctgaggccgttgccTGTAGGCTGGAGCTGCTTC	
abmA2-Del-F	ctggccgaggccgacgaggccgaactcgcccgcgcgggaATTCCGGGGATCCGTCGACC	For disrupting *abmA2*
abmA2-Del-R	gtccaggccgggcacggtgtcggcggcggtgaagccgtgTGTAGGCTGGAGCTGCTTC	
abmA3-Del-F	tcccacgacggcttcgtcgcactcctccgcgaggaagtgATTCCGGGGATCCGTCGACC	For disrupting *abmA3*
abmA3-Del-R	gcgcaccgccacggcgtagaggtgctccaggctcggtgcTGTAGGCTGGAGCTGCTTC	
abmA4-Del-F	cacctggacaccgaggtcgacatgaccgccgtcctcgccATTCCGGGGATCCGTCGACC	For disrupting *abmA4*
abmA4-Del-R	ctccaggccggccttgatctcggcgaggacgtccgcggcTGTAGGCTGGAGCTGCTTC	

abmA5-Del-F	ggcgaccggctcgccgaggaggacgacctcctgggcgccATTCCGGGGATCCGTCGACC	For disrupting *abmA5*
abmA5-Del-R	caggctggtctcggtcaggtggcggggtgagccgtggacTGTAGGCTGGAGCTGCTTC	
abmT-Del-F	ctcttctgcttccaccacgccggcggcaccgcctcggtcATTCCGGGGATCCGTCGACC	For disrupting *abmT*
abmT-Del-R	ctgggcgtccctgatgaacaggtgtccgccgggcacggtTGTAGGCTGGAGCTGCTTC	
abmB1-Del-F	tacaagaccgctgcccctaccgctccgaccggggacagcATTCCGGGGATCCGTCGACC	For disrupting *abmB1*
abmB1-Del-R	cgccgtgtcgctgccgccctcgggtcgctggggcagcggTGTAGGCTGGAGCTGCTTC	
abmH-Del-F	gcttccctggactgccgattggtgctcgcggcctcccgtATTCCGGGGATCCGTCGACC	For disrupting *abmH*
abmH-Del-R	gaggcaggagaggtcgaggccgtcggtcggggggacgttTGTAGGCTGGAGCTGCTTC	
orf(+1)-Del-F	cttgacgcggtggtcagattgggcctgttggcagaaataATTCCGGGGATCCGTCGACC	For disrupting *orf(+1)*
orf(+1)-Del-R	cgggagctgatgagccgactggaagagtccctgtcggtaTGTAGGCTGGAGCTGCTTC	
orf(+2)-Del-F	cgcggccttctgcgggcttcccatctcggacaggagctcATTCCGGGGATCCGTCGACC	For disrupting *orf(+2)*
orf(+2)-Del-R	gacatcacggagagcacaccggggtacgtggccgcgctcTGTAGGCTGGAGCTGCTTC	
orf(+3)-Del-F	gctgatgaagaccgacgggtcgaggacgccggaggcgaaATTCCGGGGATCCGTCGACC	For disrupting *orf(+3)*
orf(+3)-Del-R	gtcgcctcctgcgggctgtgcggcaccgatctccacatcTGTAGGCTGGAGCTGCTTC	
orf(-1)-TF	TGCCCAAACGGCTCCTCA	For verifying the disruption of *orf(-1)*
orf(-1)-TR	CCGGTGCCCTTCTTCTCCAC	
abmI-TF	TTTGATCTGTTTCCGATGACGC	For verifying the disruption of *abmI*
abmI-TR	CGAGAAGTGGTGCTCCGTGA	
abmF3-TF	GCGTCGAAGACGAGGTGGG	For verifying the disruption of *abmF3*
abmF3-TR	CGGACATCCTGCGCTACTGG	
abmF2-TF	ATCACCTTGATCCAGTCGGTGCCGG	For verifying the disruption of *abmF2*
abmF2-TR	CCGGCACCGACTGGATCAAGGTGAT	
abmF1-TF	GAGGACGACGAGGACCGC	For verifying the disruption of *abmF1*
abmF1-TR	GCCGAGACTCTGGCCGAG	
abmD-TF	CACCTCCGACTGGTTCACGC	For verifying the disruption of *abmD*
abmD-TR	AGTTCACCTTGCCGGTCTCG	
abmA1-TF	ACGGTTAGGCGCAGGGTTAGA	For verifying the disruption of *abmA1*
abmA1-TR	AGCAGGTGCTGGGTGAGGGA	
abmA2-TF	GCCGGATGACCGAACGTATCGT	For verifying the disruption of *abmA2*
abmA2-TR	AGGTGGACGGGTGCGAAGGA	

abmA3-TF	CGCCGAGTTCTACCCAAGG	For verifying the disruption of *abmA3*
abmA3-TR	CGGTAGTGGTCGATCCTGTCC	
abmA4-TF	GGCTTCGTCGCACTCCTCC	For verifying the disruption of *abmA4*
abmA4-TR	CCCACTGTTCCTTGATGCTGAC	
abmA5-TF	CGTCCTGCGGCTCAACCT	For verifying the disruption of *abmA5*
abmA5-TR	TCCCAGCCCTTGTAGACCG	
abmT-TF	TCATCCCGCTGGAGGACTGG	For verifying the disruption of *abmT*
abmT-TR	GGAGACGGAGGCGAAGGAG	
abmB1-TF	AGCCAGTACGGCGCATTCC	For verifying the disruption of *abmB1*
abmB1-TR	TCAGCGAGTCGAAGCCCAGTT	
abmH-TF	ACATTCGTCTGAGGGAACACCG	For verifying the disruption of *abmH*
abmH-TR	GACCACAGAGCAACGGGAGC	
orf(+1)-TF	CGTCTACCGCAAACTCAACATC	For verifying the disruption of *orf(+1)*
orf(+1)-TR	TTCCGCATTGGCACTGGA	
orf(+2)-TF	CGTTTCCAGTGCCAATGCG	For verifying the disruption of *orf(+2)*
orf(+2)-TR	GCGGGAGAAGACGGACGAT	
orf(+3) -TF	ACAGCCTCCGCGTGATTCTT	For verifying the disruption of *orf(+3)*
orf(+3) -TF	CGTCCACCTGACCACCTTCGT	
com-abmI-F	AAATTTCCCATATGGTGCCCGCCGCCACTCAGTAC (*Nde*I sites underlined)	For cloning, and overexpression of *abmI*
com-abmI-R	ATATCGTAACTAGTTCACACCGCCTGGCGTAGC (*Spe*I sites underlined)	
com-abmH-F	AAATTTCCCATATGgtggaggctctgggcgcgatc (*Nde*I sites underlined)	For cloning and overexpression of *abmH*
com-abmH-R	TTATATACTAGTtcacgccgccgcccgggtc (*Spe*I sites underlined)	

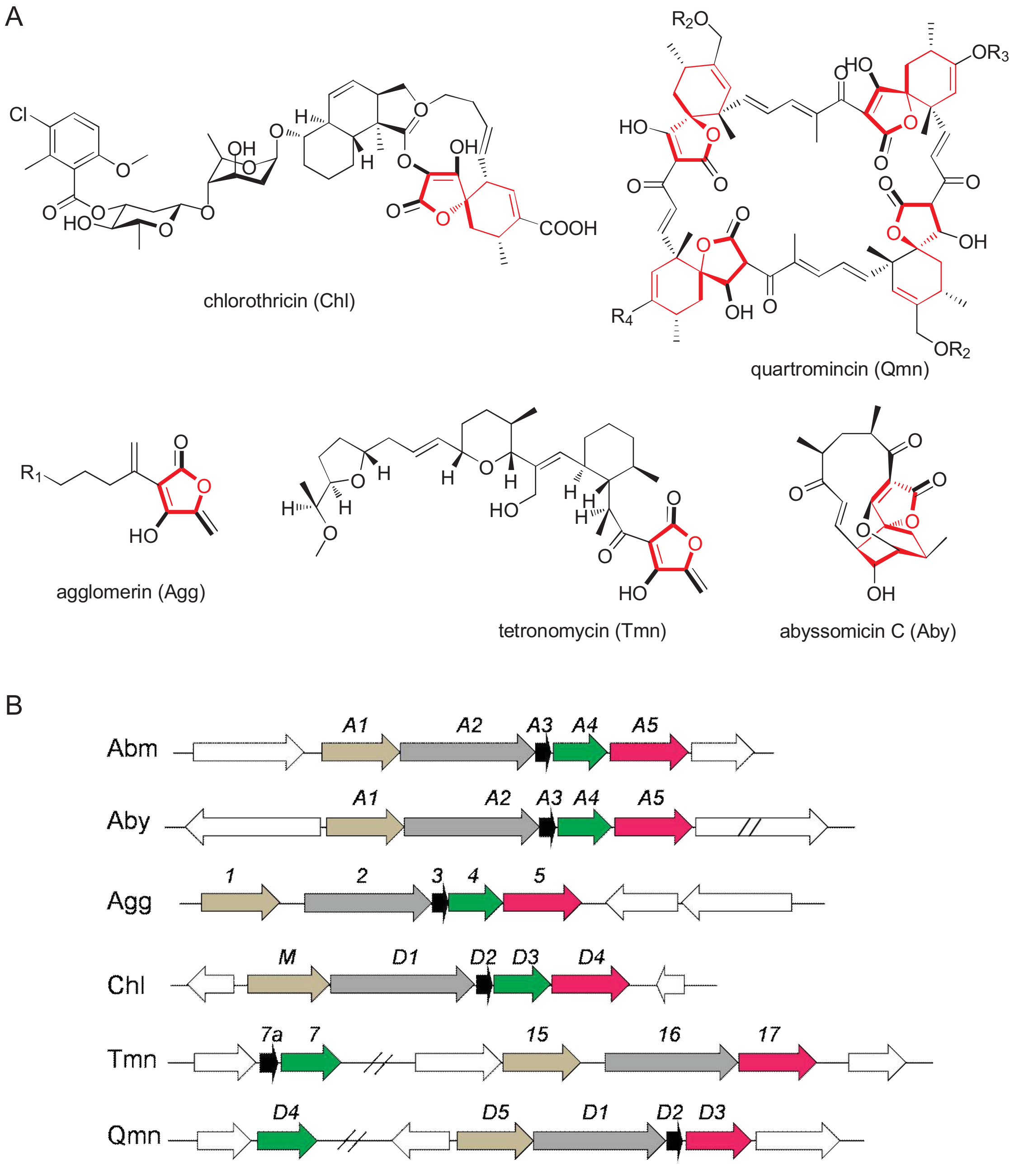

Fig. S1 Chemical structures of tetronate-containing natural products (A) and the unique set of five highly conserved genes responsible for tetronate biosynthesis (B).

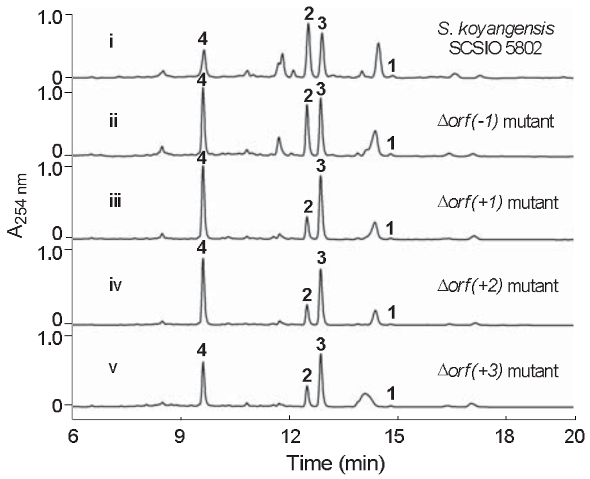

Fig. S2 HPLC analyses of fermentation extracts. i: wild-type; ii: Δ*orf(-1)* mutant; iii: Δ*orf(+1)* mutant; iv: Δ*orf(+2)* mutant; v: Δ*orf(+3)* mutant.

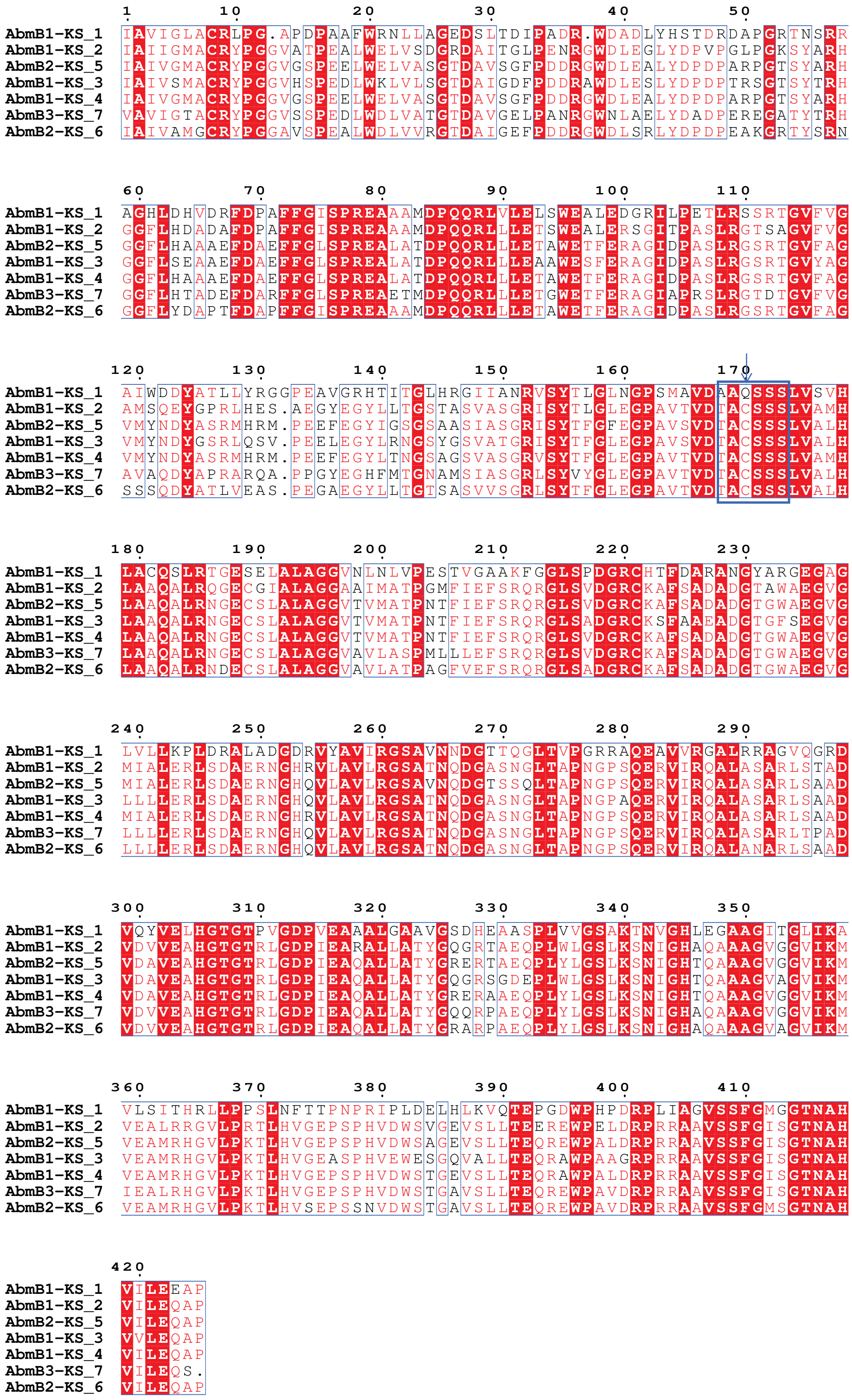

Fig. S3 Alignments of seven KS domains of AbmB1-B3. The conserved motif of KS domains is indicated by red box. The vertical arrow indicates the relacement of the transthioesterification site Cys (C) of AbmB1-KS_1 with Gln (Q).

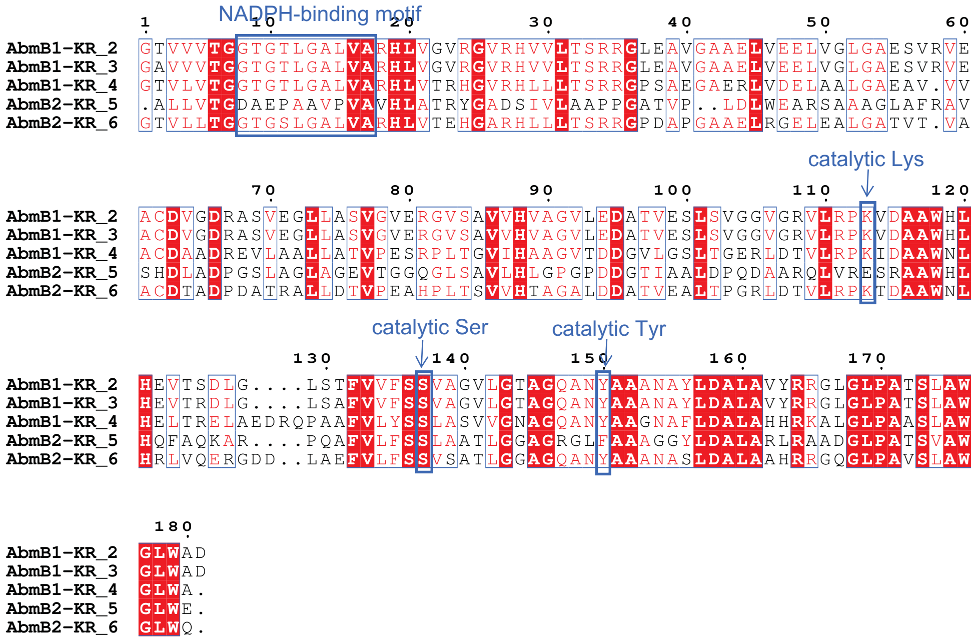

Fig. S4 Alignments of five KR domains of AbmB1-B2. The NADPH-binding motif of KR domains is indicated by blue box. The vertical arrow indicates the active catalytic amino acids.

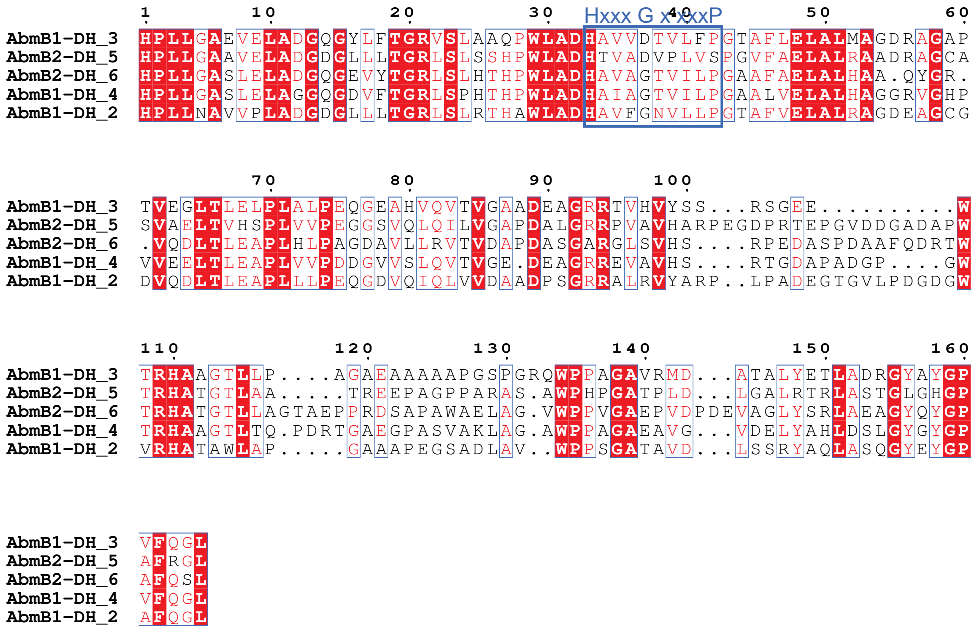

Fig. S5 Alignments of five DH domains of AbmB1-B2. The conserved mitif HxxxGxxxxP motif is indicated by blue box.

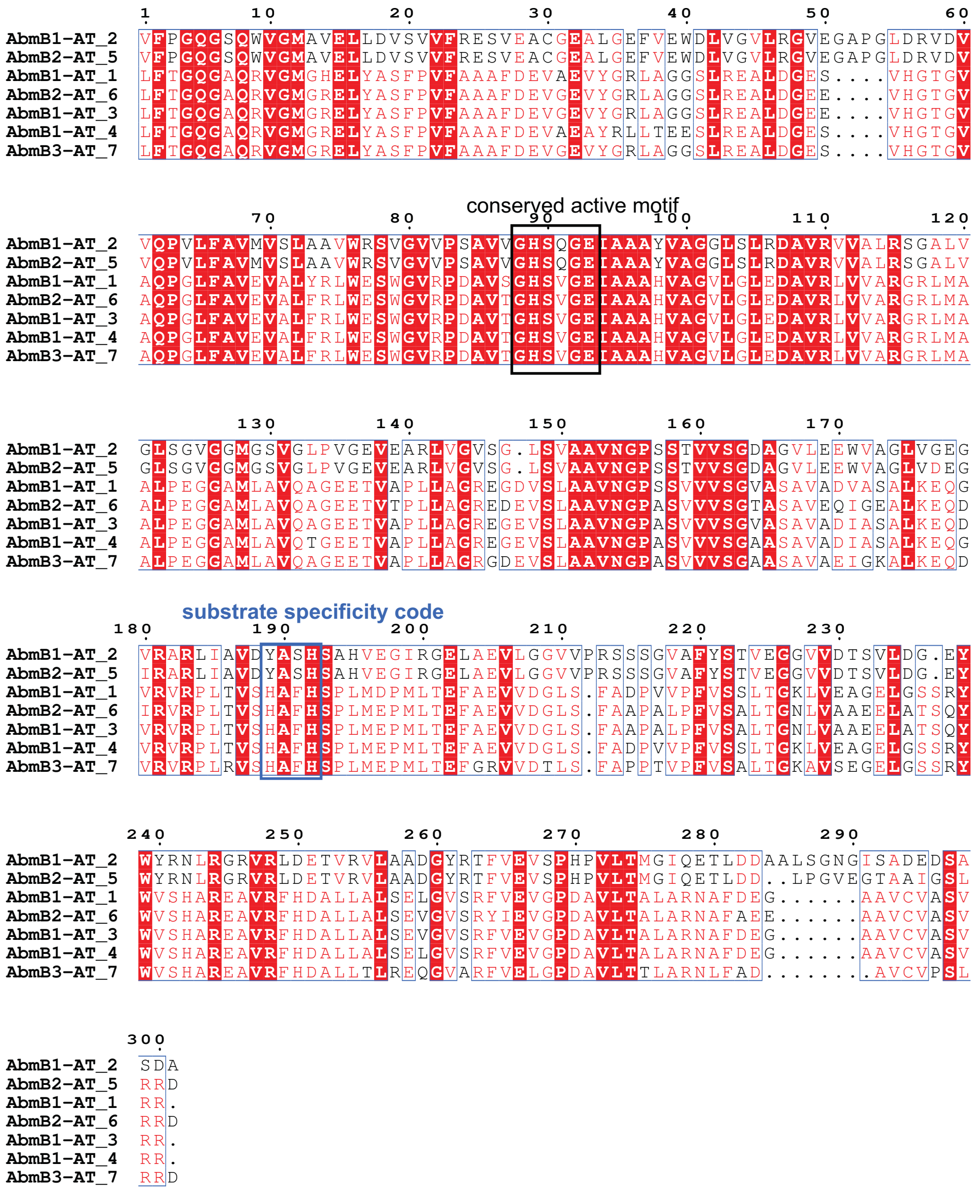

Fig. S6 Alignments of seven AT domains of AbmB1-B3. The conserved active motif of AT domains is indicated by black box. The red box indicates the motif for the substrate specifity.

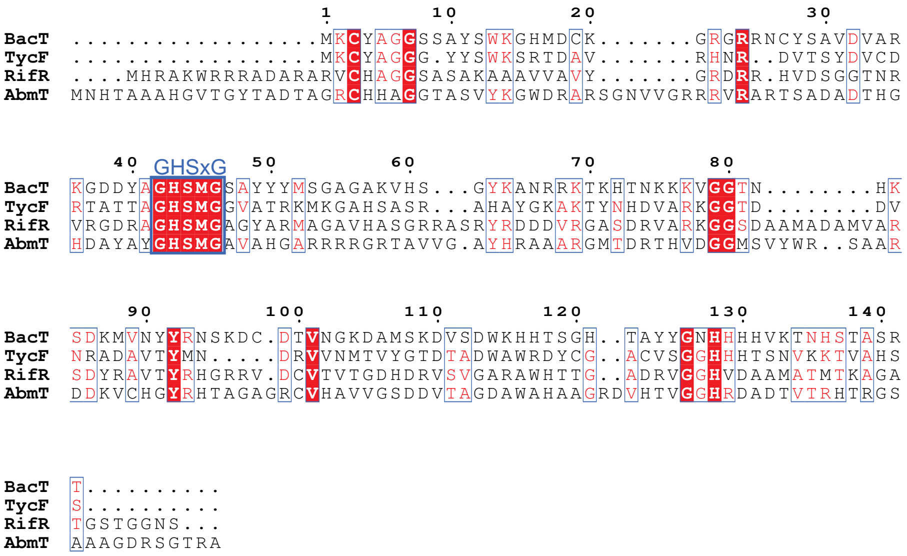

Fig. S7 Alignments of AbmT with the typical type II TEs. The following typeII TEs are used for amino acid alighments: BacT from *Bacillus licheniformis* (AF007865.2); TycF from *Brevibacillus brevis* (AF004835); RifR from *Amycolatopsis mediterranei S699* (AF040570).The conserved GHSxG motif of type II TEs is indicated by blue box.

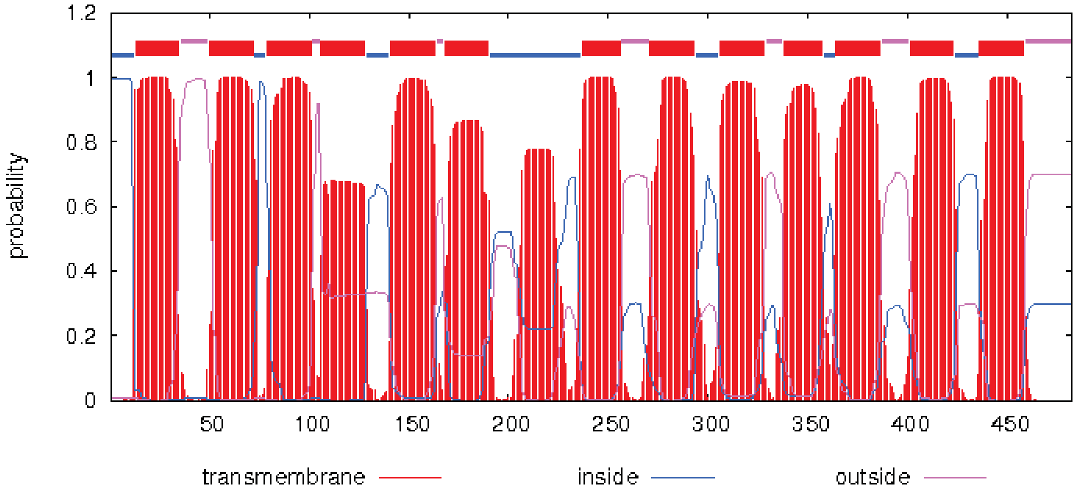

Fig. S8 The 14 transmembranes helixs of AbmD predicted by HMMTOP pogram.

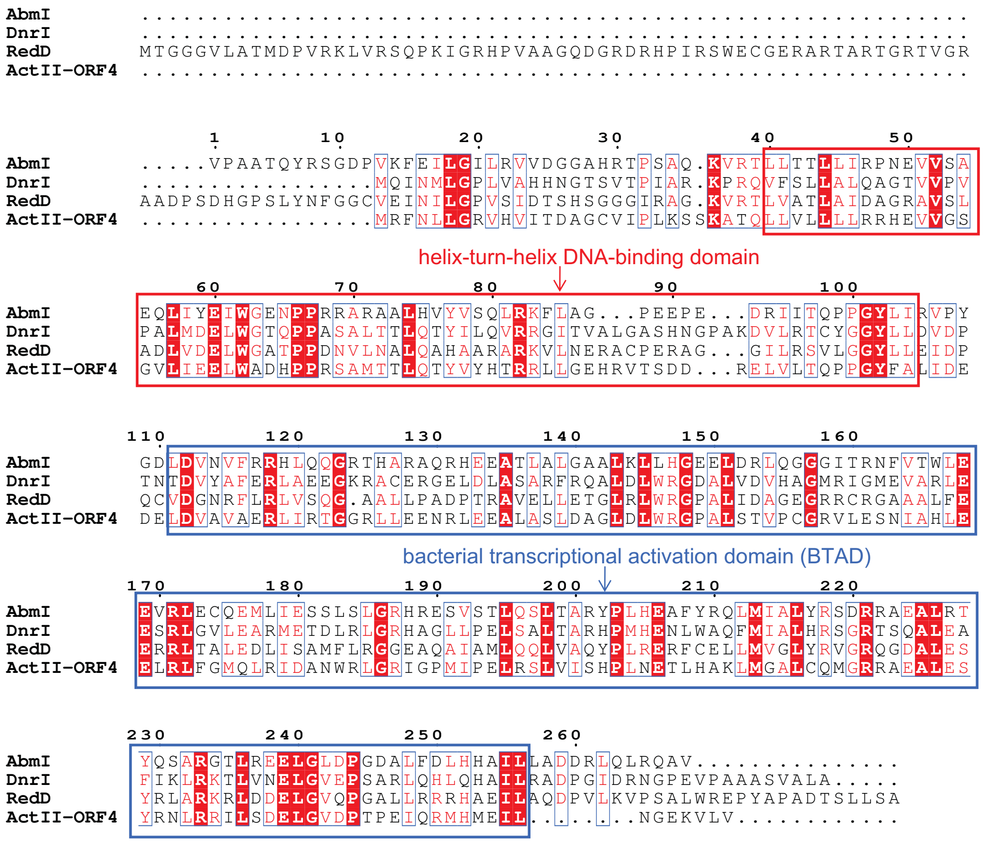

Fig. S9 Alignments of AbmI with the chacterized SARP regulators. The following regulators are used for amino acid alighments: DnrI from *Streptomyces peucetius* (AAA26736.1); RedD from *Streptomyces coelicolor* A3(2) (AAA88556.1); ActII-ORF4 from *synthetic construct* (AAK32147.1). The conserved HTH DNA-binding domian is indecated by red box, and the conseved BTAD is indecated with blue box.

Walker A

G XX XXG K[T/S]

```
AbmH   ....VEALGAIRDGRRVVELVGDPGTGKTRLLTQLAAEARSRGLVVAQGRATEGDQRVML
AveR   ..MQGVSCLHPPRKPEELTLVDRETQFRALRLALTECAAGTVKLLVAEGGMGCGKSTFLG
GdmRI  MTAEINSSLRRNPPQRGLMSMGLTDYQSRLRDVLGTAARGNGGLLQVVGGPGVGKATLLS
NysRI  ......MRKQSGSSGLLTTLVGRDDELRTLARHAAAARDGRAGLVLLHGPAGMGKTSLLR
PikD   ................MNLVERDGEIAHLRAVLDASAAGDGTLLLVSGPAGSGKTELLR
LuxR   ............................................................

AbmH   KSITASLDCRLVLAASRDLPNDAAATIRAIWSQSPVRFGEAFGGDGDHESVPGVAGREP.
AveR   EALHTAAASGFAVLRAAGLPADHRQPLGVLQQLLNDPAPEDTARTAVRPMPVQHVRGA..
GdmRI  SLEEQAAESDAVCLTASGFAEDTAIPFNIVEQLIRSSAAMGELDVVARWRTAGERYSEAE
NysRI  SFTASDVCRGMTVLYGTCGETVAGAGYGGVRELLGGLGLSGGDARRSPLLEGLAARALPA
PikD   SLRRLAAERETPVWSVRALPGDRDIPLGVLCQLLRSAEQHGADTSAVRDLLDAASRRAGN
LuxR   ............................................................
```

Walker B

h h h hD

```
AbmH   .........RDLVLSAMRGLLERLAG....NSPTVVILDDSHWADPYTTDLIDQLVRYPL
AveR   ....................LERLAA....GAPLAIGIDDVQDADPESLHCLMRLTRHSP
GdmRI  HG......MLAMLVREISDVLHRIAG....GKQLVIAVDDVEHVDYPSLMCLLHIARHAS
NysRI  LTADPAGPDAATGAYPVLHGLYWLAARLMAQRPLVLVLDDVHWCDERSLAWIDFLLRRAE
PikD   LTSPADAPLRVDETHRLHDWLLSVSR....RTPFLVAVDDLTHADTASLRFLLYCAAHHD
LuxR   ............................................................

AbmH   NAPLLIVLAHRPRQTGSTLRSTLAQGVVSGVVERIQLNELTLGQAADLLS....VEARSR
AveR   TSRILLLCTALACSP.AADPVLEAELMRQTAFERITLDCLSLDGVTGLVSDRCARPTAPP
GdmRI  G.....TRTLVAMSS.GRTHHLCARVQGFHNLYKVEIGTLSESGVVRLLERHADADLADR
NysRI  DLPLLVVLAWRSEAE.PVAPAVLADIAAQRRPTVLGLHPLGPDDIGEMVRRVFRTTAAPS
PikD   QGGIGFVMTERASQR.AGYRVFRAELLRQPHCRNMWLSGLPPSGVRQLLAHYYGPEAAER
LuxR   ............................................................

AbmH   RSVDLYRESQGVPQYLLALANP.............................GDNDEFVQL
AveR   PADYCLTVTGGNPLLLRALLEEHSEADAPSAPRPAEPSALHSPPQAAPPRPVVGGRFYQS
GdmRI  IGASCHAISGGNPRLVRALLHDHLMS.................APDGSEREVTVGAEFRD
NysRI  FVSRVAAVSGGNPLALARLLDELRAEG............VRPDAAGERRAEVGSHVLARS
PikD   RAPAYHATTGGNPLLLRALTQD..........RQASHTTLG...AAGGDEPVHGDAFAQA
LuxR   ............................................................

AbmH   VQTEAALLGDDERRVLEAAAVIDGCFDVTALASVAGLERARVCVAATRLLNRDLFRHATP
AveR   VLACLSRTETAIRQTAGALAVLGGRARADLLPQLLGASPASVTRGLRALEATGLTTS...
GdmRI  SYRWSLLCHPSLLQMAQILAVLGPYGSPRRVAGLLECGEERAARAIDVLDSAGLLED...
NysRI  VRCLLERRPPWVRGVARAIAVLG.PECTELLAALAGVPAATVDEALLVLRRAGILAADR.
PikD   VLDCLHRSAEGTLETARWLAVLE.QSDPLLVERLTGTTAAAVERHIQELAAIGLLDED..
LuxR   ............................................................

AbmH   ASVMVLRHPLVRDAVYAATDGCWRREAHRRAIDYLRDCGAPPSELAHHVAAAPDSSDSDD
AveR   ...GRFRHPVAEAAALDALDPSRRAHLHRRAAALQHHDGAAPRDVARHLLAARHAAGPWA
GdmRI  ...GCFRHPAARSVALETLAVEERARLGAKAAELLYADGADPIAVAELLVTAGKTPDHKG
NysRI  ...VDFVHDVVRSAVLDDVAPPTLAELRTNAALLLSDAGRPSEELAGQLMLLPVLDQPWM
PikD   ...GTLGQPAIREAALQDLPAGERTELHRRAAEQLHRDGADEDTVARHLLVGGAPDAPWA
LuxR   ............................................................

AbmH   LDILLRAAEEPLP.GNPERAITWLEAALRLSLPRRQRGLALIALSRALCVAERFADAMPH
AveR   VSVLRDAAEQSLAQDDVASAVSCLELAYGACVRERERPEIRIRLAAAFGRTN.IAVAEEH
GdmRI  VAVLWHAAQKNLDEGRTEEAVAGLRLAARADLGRERLDIRMALVGALWAGN.PAAAEPE
NysRI  AAVLRDAAAQAESRGAPEAGVRCLYRVLEVEPDN...VAVRIQMARALAEIN.PPEAMRL
PikD   LPLLERGAQQALFDDRLDDAFRILEFAVRSSTDNTQLARLAPHLVAASWRMN.PHMTTRA
LuxR   ............................................................

AbmH   LREAVAMGAAL..PVEIWAKAVSQSAAIDTLFGRYSEANATLDGALAMLRGRPSLEEVEL
AveR   LADLVATLREG.ELTG...HQTALLVPLLVNHGRLGEAREAMDRLNAADDARGLCADGGF
GdmRI  LDRLMAAIRTE.SPADIPEQYLCFLLFMVLWFGRFSDVEEAFTWLSGSGGSGNASSMAAL
NysRI  LKEALSLAGDVRTRAQVAVQYGFTCLAVQESPSGVRMLEDALAELTAELGPEPGPVDREL
PikD   LA.LFDRLLSG.ELPPS..HPVMALIRCLVWYGRLPEAADALSRLRPSSDNDALELS...
LuxR   ............................................................

AbmH   LVRRGVIGLYDGGALDTGTVDLAARRAQEFACHTSTAAAMALR.........ALKSTSLG
AveR   PMAAPWPSTAHLAARRDPAARRDPGTRRDPADKPFLPRQSGAPQPRPEDGRGQQPTAALW
GdmRI  RVTRQWVTFLKPTLIHHFPDQDPSDREVDGLWAANLEHGLQLS........VDELGMEIG
NysRI  RTLVESVLLIVGADEKVTIGAVRDRAARLTMPPGDTPAQR...........QMLAMTTVL
PikD   .LTRMWLAALCPPLLESLPATPEP..ERGPVPVRLAPRTT...........ALQAQAGVF
LuxR   ............................................................
```

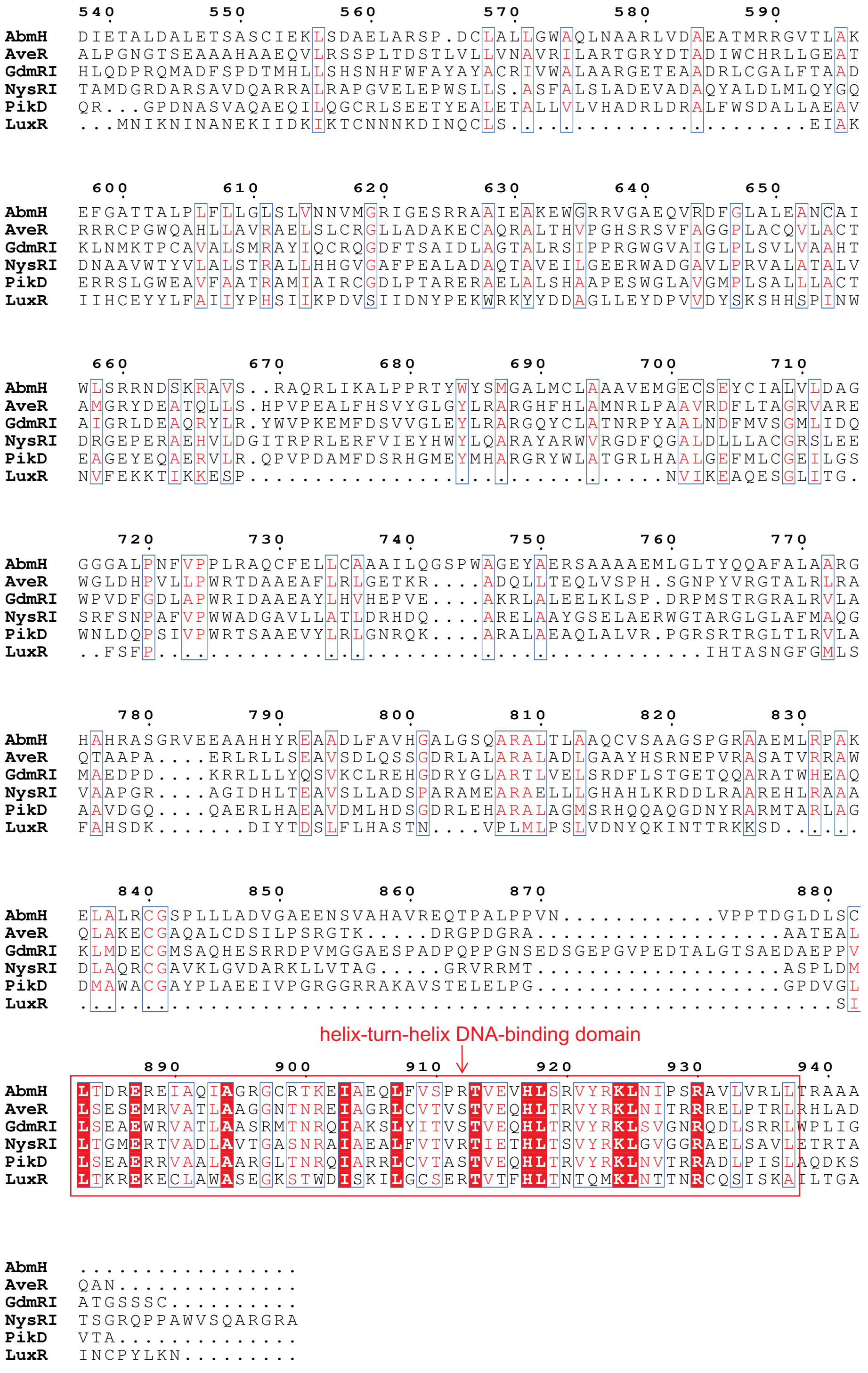

Fig. S10 Alignments of AbmH with the characterized LuxR-regulators from *Streptomyces avermitilis* (AveR, BAA84600.1) , *Streptomyces hygroscopicus* (GdmRI, ABI93791.1), *Streptomyces noursei* ATCC 11455 (NysRI, AAF71778.1), *Streptomyces venezuelae* (PikD, AAC68887.1) and *Vibrio fischeri* ES114 (LuxR, YP_206883.1). The conserved Walker A motif (GxxxxGK[T/S]) and Walker B motif (hhhhD) (where h is a hydrophobic residue) in the NTP-binding domain is indecated by blue box; the conserved HTH DNA-binding domian is indecated with red box.

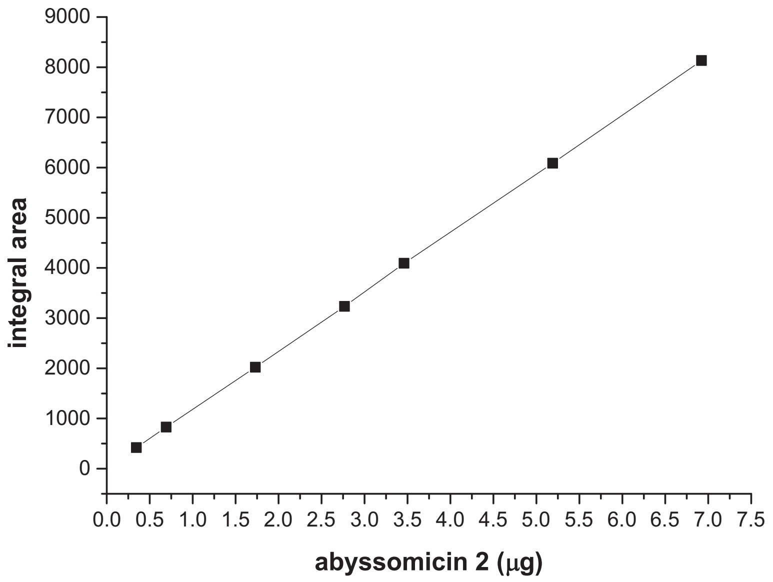

Fig. S11 The quantitative HPLC standard curve for abyssomicin 2 (**3**). The curve was generated via analysis of a concentration gradient of 0.346 μg, 0.692 μg, 1.730 μg, 2.768 μg, 3.461 μg, 5.191 μg, 6.922 μg of **3**. UV absorptions were maintained below 1 A unit to ensure appropriate confidence of the generated standard curve and avoid deviation from linearity.

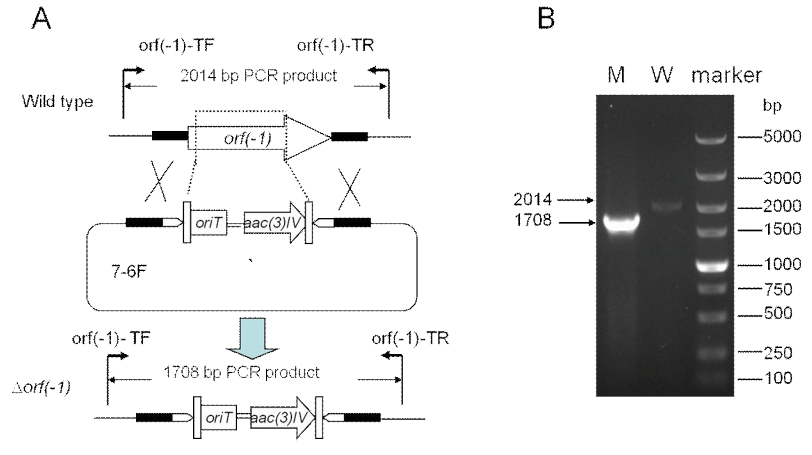

Fig. S12 Disruption of *orf(-1)* in wild-type *S. koyangensis* SCSIO 5802 via PCR-targeting. (A) Schematic representation for disruption of *orf(-1)*. (B*)* PCR analyses of the wild-type strain and the of *orf(-1)* double-cross mutant carried out using the primers listed in Table S2. Marker: DNA molecular ladder; W: using the genomic DNA of *S. koyangensis* SCSIO 5802 as template; M: using the genomic DNA of *orf(-1)* mutant as template.

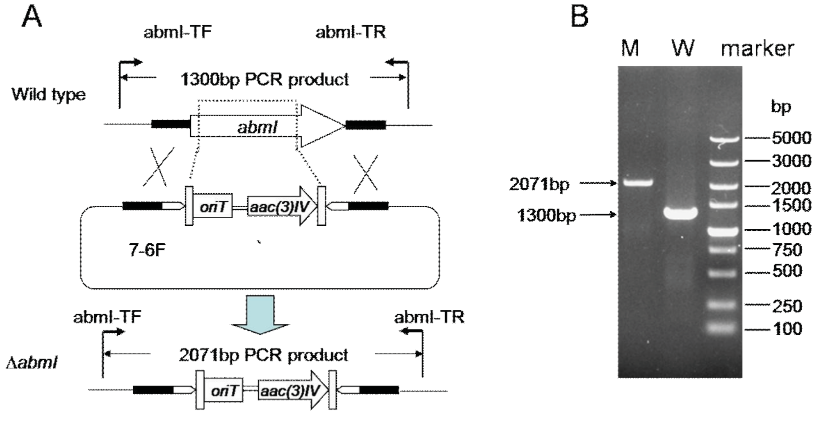

Fig. S13 Disruption of *abmI* in wild-type *S. koyangensis* SCSIO 5802 via PCR-targeting. (A) Schematic representation for disruption of *abmI.* (B*)* PCR analyses of the wild-type strain and the of *abmI* double-cross mutant carried out using the primers listed in Table S2. Marker: DNA molecular ladder; W: using the genomic DNA of *S. koyangensis* SCSIO 5802 as template; M: using the genomic DNA of *abmI* mutant as template.

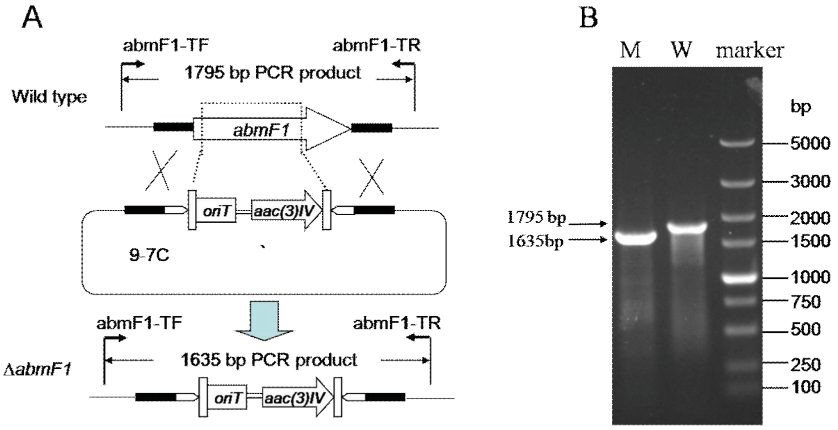

Fig. S14 Disruption of *abmF1* in wild-type *S. koyangensis* SCSIO 5802 via PCR-targeting. (A) Schematic representation for disruption of *abmF1*. (B*)* PCR analyses of the wild-type strain and the of *abmF1* double-cross mutant carried out using the primers listed in Table S2. Marker: DNA molecular ladder; W: using the genomic DNA of *S. koyangensis* SCSIO 5802 as template; M: using the genomic DNA of *abmF1* mutant as template.

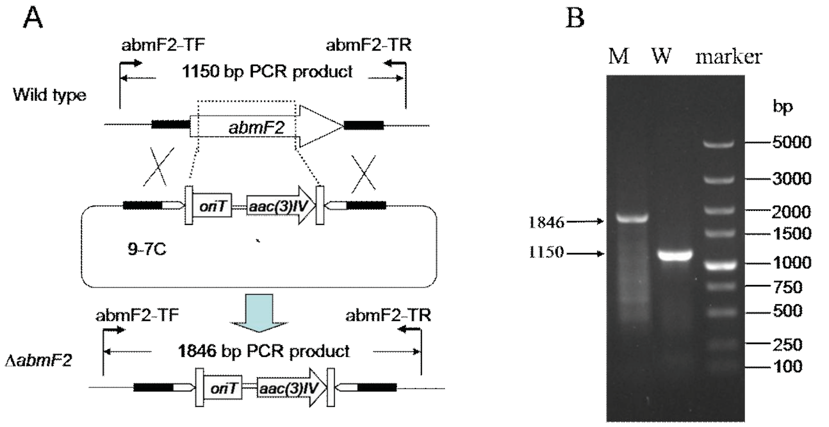

Fig. S15 Disruption of *abmF2* in wild-type *S. koyangensis* SCSIO 5802 via PCR-targeting. (A) Schematic representation for disruption of *abmF2*. (B*)* PCR analyses of the wild-type strain and the of *abmF2* double-cross mutant carried out using the primers listed in Table S2. Marker: DNA molecular ladder; W: using the genomic DNA of *S. koyangensis* SCSIO 5802 as template; M: using the genomic DNA of *abmF2* mutant as template.

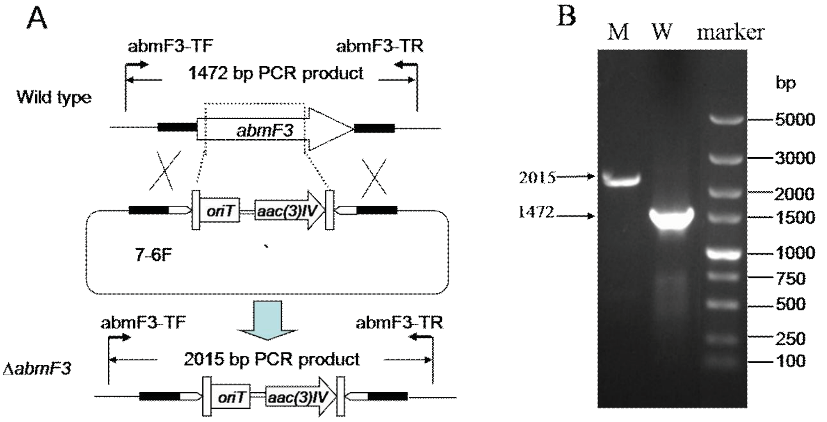

Fig. S16 Disruption of *abmF3* in wild-type *S. koyangensis* SCSIO 5802 via PCR-targeting. (A) Schematic representation for disruption of *abmF3*. (B*)* PCR analyses of the wild-type strain and the of *abmF3* double-cross mutant carried out using the primers listed in Table S2. Marker: DNA molecular ladder; W: using the genomic DNA of *S. koyangensis* SCSIO 5802 as template; M: using the genomic DNA of *abmF3* mutant as template.

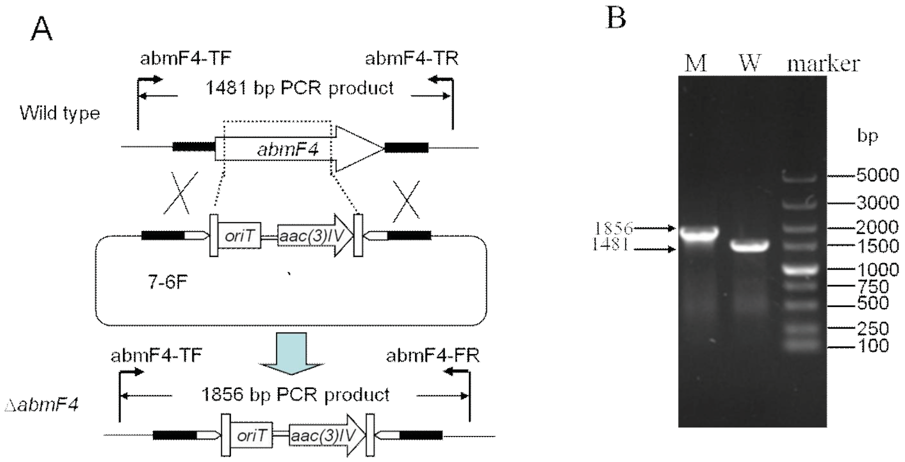

Fig. S17 Disruption of *abmF4* in wild-type *S. koyangensis* SCSIO 5802 via PCR-targeting. (A) Schematic representation for disruption of *abmF4*. (B*)* PCR analyses of the wild-type strain and the of *abmF4* double-cross mutant carried out using the primers listed in Table S2. Marker: DNA molecular ladder; W: using the genomic DNA of *S. koyangensis* SCSIO 5802 as template; M: using the genomic DNA of *abmF4* mutant as template.

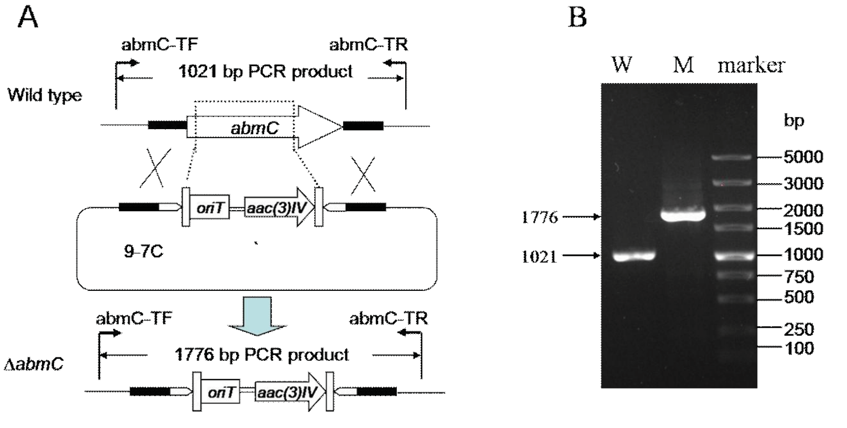

Fig. S18 Disruption of *abmC* in wild-type *S. koyangensis* SCSIO 5802 via PCR-targeting. (A) Schematic representation for disruption of *abmC.* (B*)* PCR analyses of the wild-type strain and the of *abmC* double-cross mutant carried out using the primers listed in Table S2. Marker: DNA molecular ladder; W: using the genomic DNA of *S. koyangensis* SCSIO 5802 as template; M: using the genomic DNA of *abmC* mutant as template.

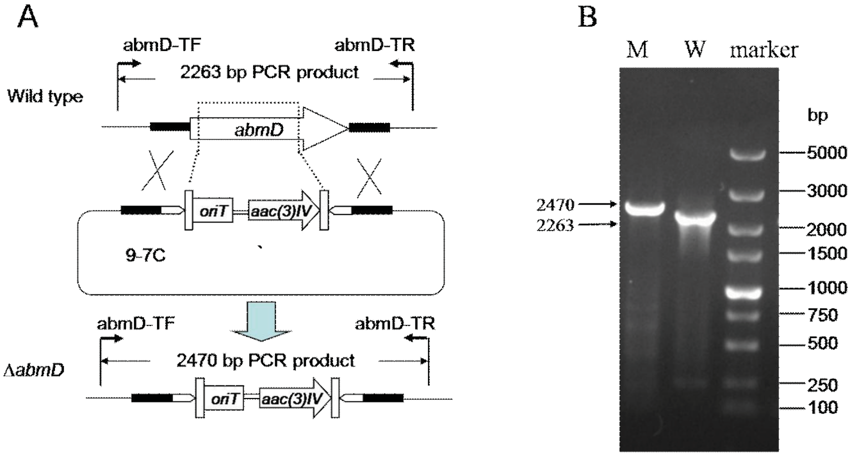

Fig. S19 Disruption of *abmD* in wild-type *S. koyangensis* SCSIO 5802 via PCR-targeting. (A) Schematic representation for disruption of *abmD.* (B*)* PCR analyses of the wild-type strain and the of *abmD* double-cross mutant carried out using the primers listed in Table S2. Marker: DNA molecular ladder; W: using the genomic DNA of *S. koyangensis* SCSIO 5802 as template; M: using the genomic DNA of *abmD* mutant as template.

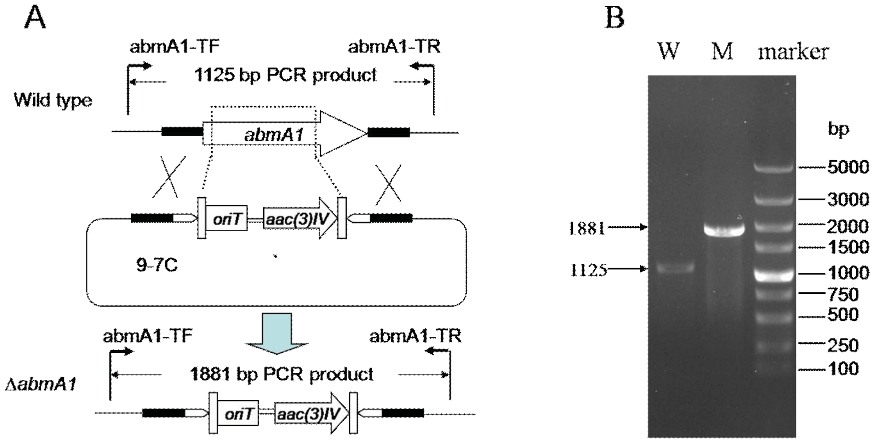

Fig. S20 Disruption of *abmA1* in wild-type *S. koyangensis* SCSIO 5802 via PCR-targeting. (A) Schematic representation for disruption of *abmA1*. (B*)* PCR analyses of the wild-type strain and the of *abmA1* double-cross mutant carried out using the primers listed in Table S2. Marker: DNA molecular ladder; W: using the genomic DNA of *S. koyangensis* SCSIO 5802 as template; M: using the genomic DNA of *abmA1* mutant as template.

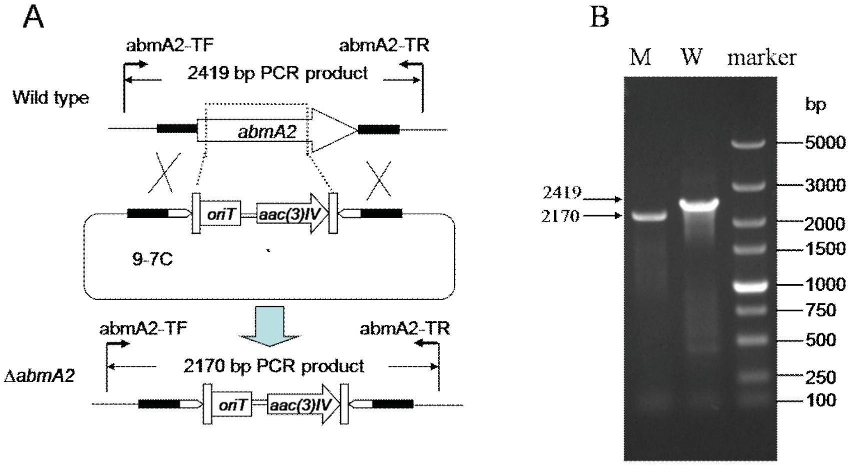

Fig. S21 Disruption of *abmA2* in wild-type *S. koyangensis* SCSIO 5802 via PCR-targeting. (A) Schematic representation for disruption of *abmA2*. (B*)* PCR analyses of the wild-type strain and the of *abmA2* double-cross mutant carried out using the primers listed in Table S2. Marker: DNA molecular ladder; W: using the genomic DNA of *S. koyangensis* SCSIO 5802 as template; M: using the genomic DNA of *abmA2* mutant as template.

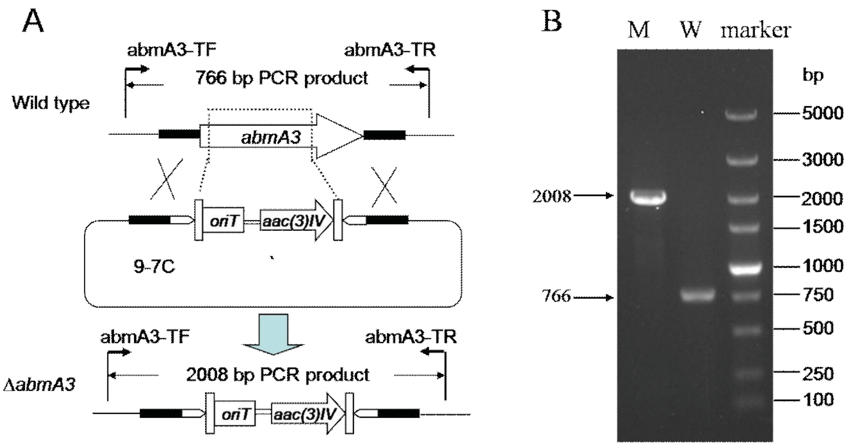

Fig. S22 Disruption of *abmA3* in wild-type *S. koyangensis* SCSIO 5802 via PCR-targeting. (A) Schematic representation for disruption of *abmA3*. (B*)* PCR analyses of the wild-type strain and the of *abmA3* double-cross mutant carried out using the primers listed in Table S2. Marker: DNA molecular ladder; W: using the genomic DNA of *S. koyangensis* SCSIO 5802 as template; M: using the genomic DNA of *abmA3* mutant as template.

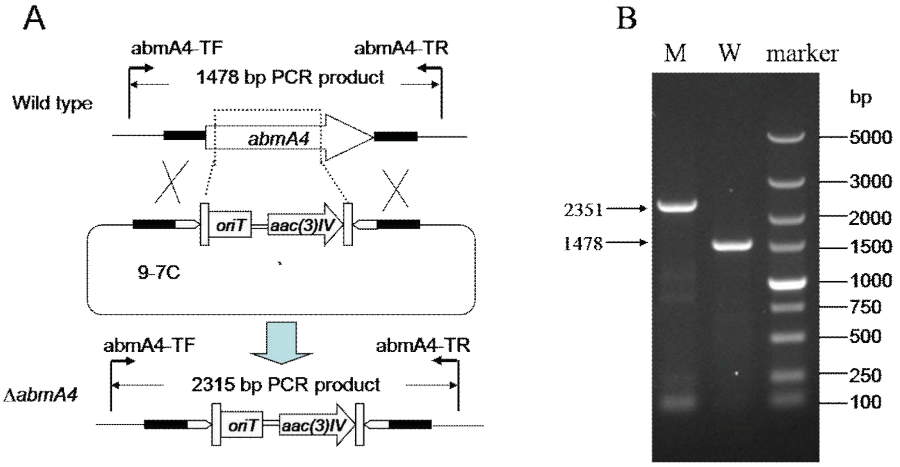

Fig. S23 Disruption of *abmA4* in wild-type *S. koyangensis* SCSIO 5802 via PCR-targeting. (A) Schematic representation for disruption of *abmA4*. (B*)* PCR analyses of the wild-type strain and the of *abmA4* double-cross mutant carried out using the primers listed in Table S2. Marker: DNA molecular ladder; W: using the genomic DNA of *S. koyangensis* SCSIO 5802 as template; M: using the genomic DNA of *abmA4* mutant as template.

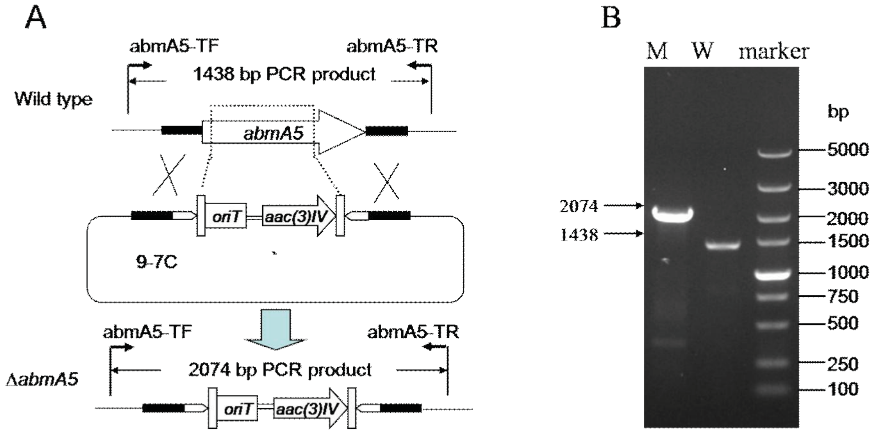

Fig. S24 Disruption of *abmA5* in wild-type *S. koyangensis* SCSIO 5802 via PCR-targeting. (A) Schematic representation for disruption of *abmA5*. (B*)* PCR analyses of the wild-type strain and the of *abmA5* double-cross mutant carried out using the primers listed in Table S2. Marker: DNA molecular ladder; W: using the genomic DNA of *S. koyangensis* SCSIO 5802 as template; M: using the genomic DNA of *abmA5* mutant as template.

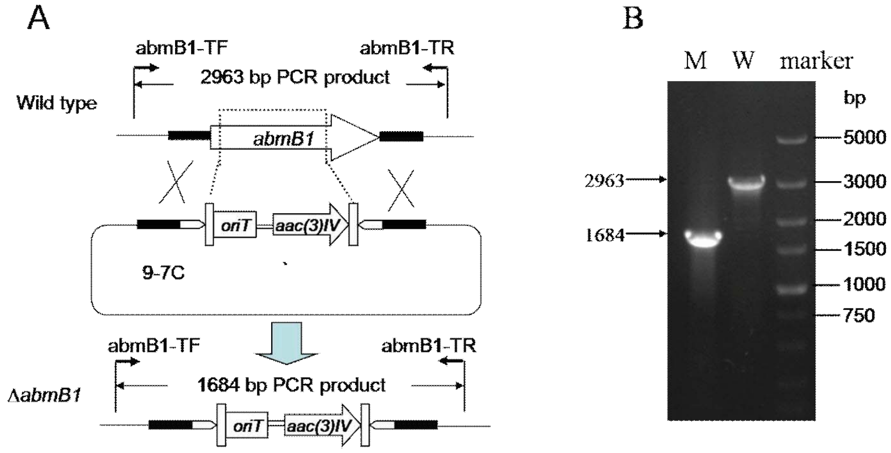

Fig. S25 Disruption of *abmB1* in wild-type *S. koyangensis* SCSIO 5802 via PCR-targeting. (A) Schematic representation for disruption of *abmB1*. (B*)* PCR analyses of the wild-type strain and the of *abmB1* double-cross mutant carried out using the primers listed in Table S2. Marker: DNA molecular ladder; W: using the genomic DNA of *S. koyangensis* SCSIO 5802 as template; M: using the genomic DNA of *abmB1* mutant as template.

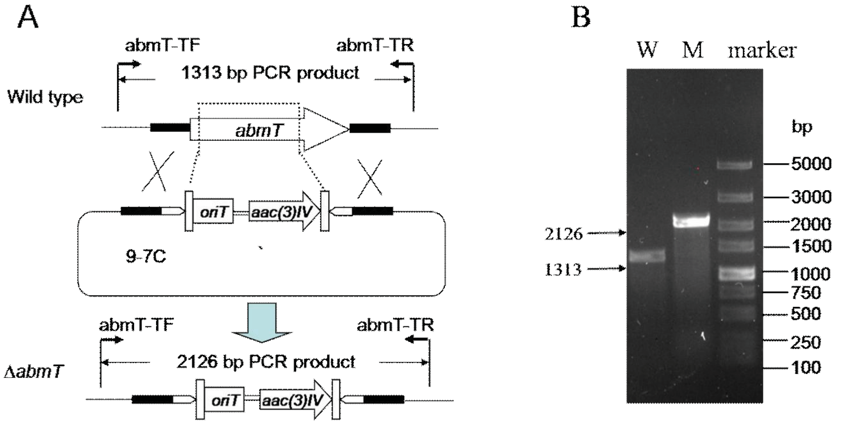

Fig. S26 Disruption of *abmT* in wild-type *S. koyangensis* SCSIO 5802 via PCR-targeting. (A) Schematic representation for disruption of *abmT.* (B*)* PCR analyses of the wild-type strain and the of *abmT* double-cross mutant carried out using the primers listed in Table S2. Marker: DNA molecular ladder; W: using the genomic DNA of *S. koyangensis* SCSIO 5802 as template; M: using the genomic DNA of *abmT* mutant as template.

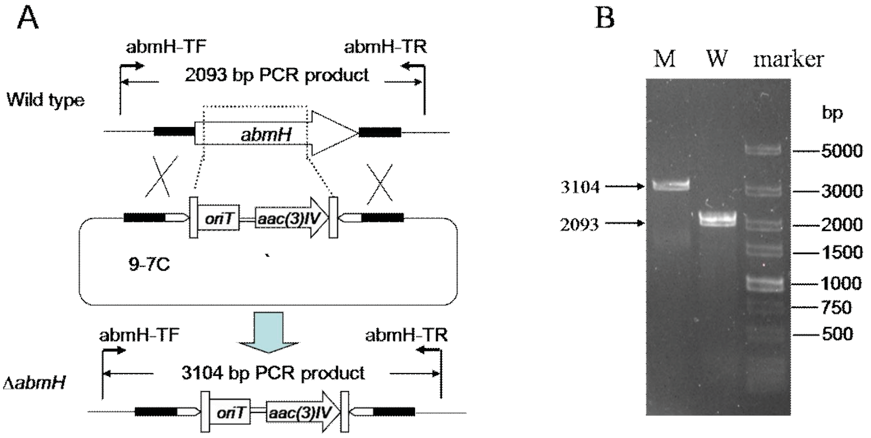

Fig. S27 Disruption of *abmH* in wild-type *S. koyangensis* SCSIO 5802 via PCR-targeting. (A) Schematic representation for disruption of *abmH.* (B*)* PCR analyses of the wild-type strain and the of *abmH* double-cross mutant carried out using the primers listed in Table S2. Marker: DNA molecular ladder; W: using the genomic DNA of *S. koyangensis* SCSIO 5802 as template; M: using the genomic DNA of *abmH* mutant as template.

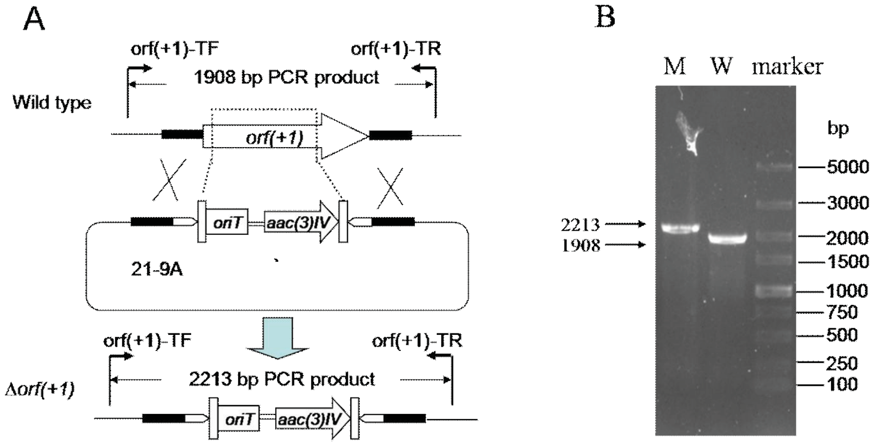

Fig. S28 Disruption of *orf(+1)* in wild-type *S. koyangensis* SCSIO 5802 via PCR-targeting. (A) Schematic representation for disruption of *orf(+1).* (B*)* PCR analyses of the wild-type strain and the of *orf(+1)* double-cross mutant carried out using the primers listed in Table S2. Marker: DNA molecular ladder; W: using the genomic DNA of *S. koyangensis* SCSIO 5802 as template; M: using the genomic DNA of *orf(+1)* mutant as template.

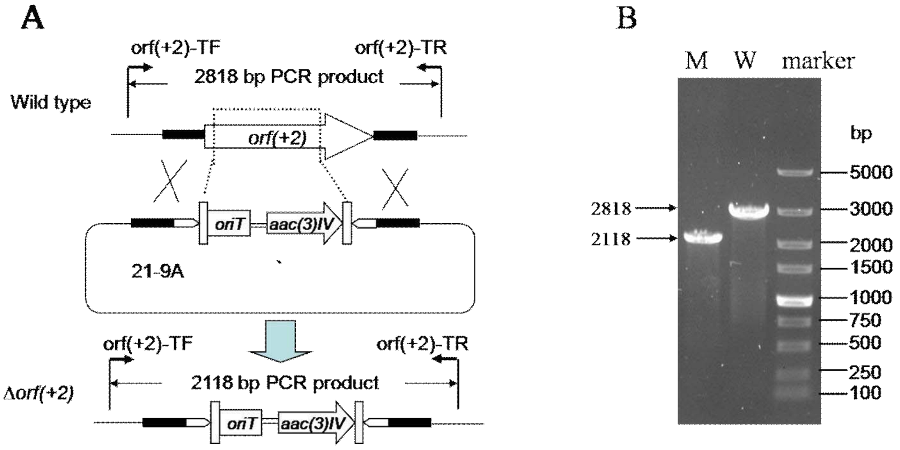

Fig. S29 Disruption of *orf(+2)* in wild-type *S. koyangensis* SCSIO 5802 via PCR-targeting. (A) Schematic representation for disruption of *orf(+2).* (B*)* PCR analyses of the wild-type strain and the of *orf(+2)* double-cross mutant carried out using the primers listed in Table S2. Marker: DNA molecular ladder; W: using the genomic DNA of *S. koyangensis* SCSIO 5802 as template; M: using the genomic DNA of *orf(+2)* mutant as template.

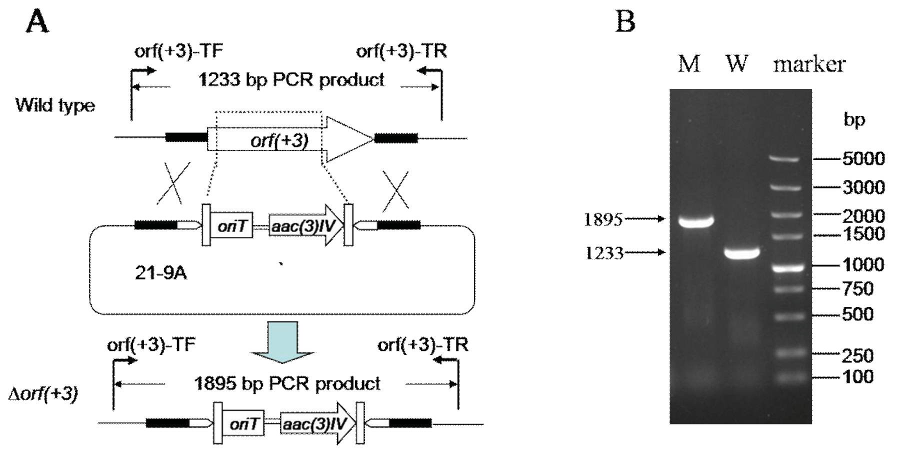

Fig. S30 Disruption of *orf(+3)* in wild-type *S. koyangensis* SCSIO 5802 via PCR-targeting. (A) Schematic representation for disruption of *orf(+3)*. (B) PCR analyses of the wild-type strain and the of *orf(+3)* double-cross mutant carried out using the primers listed in Table S2. Marker: DNA molecular ladder; W: using the genomic DNA of *S. koyangensis* SCSIO 5802 as template; M: using the genomic DNA of *orf(+3)* mutant as template.

Supplementary References

1. Datsenko KA, Wanner BL. One-step inactivation of chromosomal genes in *Escherichia coli* K-12 using PCR products. Proc Natl Acad Sci U S A. 2000;97:6640-5.
2. MacNeil DJ, Gewain KM, Ruby CL, Dezeny G, Gibbons PH, MacNeil T. Analysis of *Streptomyces avermitilis* genes required for avermectin biosynthesis utilizing a novel integration vector. Gene. 1992;111:61-8.
3. Gust B, Challis GL, Fowler K, Kieser T, Chater KF. PCR-targeted *Streptomyces* gene replacement identifies a protein domain needed for biosynthesis of the sesquiterpene soil odor geosmin. Proc Natl Acad Sci U S A. 2003;100:1541-6.
4. Hong B, Phornphisutthimas S, Tilley E, Baumberg S, McDowall KJ. Streptomycin production by *Streptomyces griseus* can be modulated by a mechanism not associated with change in the *adpA* component of the A-factor cascade. Biotechnol Lett. 2007;29:57–64.
